# Omega-3 fatty acid exposure with a low-fat diet in patients with past hypertriglyceridemia-induced acute pancreatitis; an exploratory, randomized, open-label crossover study

**DOI:** 10.1186/s12944-020-01295-7

**Published:** 2020-05-30

**Authors:** Richard L. Dunbar, Daniel Gaudet, Michael Davidson, Martin Rensfeldt, Hong Yang, Catarina Nilsson, Mats Kvarnström, Jan Oscarsson

**Affiliations:** 1 Cardiometabolic and Lipid Clinic, Corporal Michael J. Crescenz VA Medical Center, Philadelphia, PA USA; 2 Division of Translational Medicine and Human Genetics, Department of Medicine, Perelman School of Medicine at the University of Pennsylvania, Philadelphia, PA USA; 3 ICON plc, North Wales, PA USA; 4 Lipidology Unit, Community Genomic Medicine Centre and ECOGENE-21, Department of Medicine, Université de Montréal, Saguenay, QC Canada; 5 University of Chicago Pritzker School of Medicine, Chicago, IL USA; 6 Corvidia Therapeutics, Waltham, MA USA; 7 AstraZeneca, Gothenburg, Sweden

**Keywords:** Clinical trials, Fish oil, Omega-3 fatty acids, Pharmacokinetics, Triglycerides, Omega-3 ethyl esters, Omega-3 carboxylic acids, Eicosapentaenoic acid, Docosahexaenoic acid, Viscosity

## Abstract

**Background:**

Omega-3 fatty acids (OM3-FAs) are recommended with a low-fat diet for severe hypertriglyceridemia (SHTG), to reduce triglycerides and acute pancreatitis (AP) risk. A low-fat diet may reduce pancreatic lipase secretion, which is required to absorb OM3-ethyl esters (OM3-EEs), but not OM3-carboxylic acids (OM3-CAs).

**Methods:**

In this exploratory, randomized, open-label, crossover study, 15 patients with SHTG and previous AP were instructed to take OM3-CA (2 g or 4 g) and OM3-EE 4 g once daily for 4 weeks, while adhering to a low-fat diet. On day 28 of each treatment phase, a single dose was administered in the clinic with a liquid low-fat meal, to assess 24-h plasma exposure. Geometric least-squares mean ratios were used for between-treatment comparisons of baseline (day 0)-adjusted area under the plasma concentration versus time curves (AUC_0–24_) and maximum plasma concentrations (*C*_max_) for eicosapentaenoic acid (EPA) and docosahexaenoic acid (DHA).

**Results:**

Before initiating OM3-FA treatment, mean baseline fasting plasma EPA + DHA concentrations (nmol/mL) were 723 for OM3-CA 2 g, 465 for OM3-CA 4 g and 522 for OM3-EE 4 g. At week 4, mean pre-dose fasting plasma EPA + DHA concentrations increased by similar amounts (+ 735 − + 768 nmol/mL) for each treatment. During the 24-h exposure assessment (day 28), mean plasma EPA + DHA increased from pre-dose to the maximum achieved concentration by + 32.7%, + 45.8% and + 3.1% with single doses of OM3-CA 2 g, OM3-CA 4 g and OM3-EE 4 g, respectively. Baseline-adjusted AUC_0–24_ was 60% higher for OM3-CA 4 g than for OM3-EE 4 g and baseline-adjusted *C*_max_ was 94% higher (both non-significant).

**Conclusions:**

Greater 24-h exposure of OM3-CA versus OM3-EE was observed for some parameters when administered with a low-fat meal at the clinic on day 28. However, increases in pre-dose fasting plasma EPA + DHA over the preceding 4-week dosing period were similar between treatments, leading overall to non-significant differences in baseline (day 0)-adjusted AUC_0–24_ and *C*_max_ EPA + DHA values. It is not clear why the greater 24-h exposure of OM3-CA versus OM3-EE observed with a low-fat meal did not translate into significantly higher pre-dose fasting levels of DHA + EPA with longer-term use.

**Trial registration:**

ClinicalTrials.gov, NCT02189252, Registered 23 June 2014.

## Background

Acute pancreatitis is associated with increased mortality, which may be as high as 30% in patients with severe disease [1]. Hypertriglyceridemia is the third most common cause of acute pancreatitis, behind alcohol use and gallstone disease, accounting for 1–10% of all cases [2]. The aim of triglyceride (TG)-lowering therapy in patients with severe hypertriglyceridemia (SHTG) is to lower TG concentrations and maintain them below 500 mg/dL (5.6 mmol/L) to reduce the risk of acute pancreatitis [3–5]. A recent population-based study found that the incident risk of acute pancreatitis fell by 4% for every 100 mg/dL drop in the TG concentration [6]. Randomized controlled trials show that 3–4 g/day of the omega-3 fatty acids (OM3-FAs) eicosapentaenoic acid (EPA) and docosahexaenoic acid (DHA) lowers serum TG by 25–45% in patients with SHTG [7]. OM3-FAs (and fibrates and nicotinic acid) are recommended in combination with the National Cholesterol Education Program Therapeutic Lifestyle Changes (NCEP TLC) diet for lowering serum TG [3, 5, 8].

Beyond averting pancreatitis, TG lowering and lowering of non-high-density lipoprotein cholesterol (non-HDL-C) by OM3-FA formulations share the potential to reduce the residual risk of cardiovascular events in patients taking statins [9–11]. Much of this residual risk is thought to arise from residual dyslipidemia, which may be harder to assess by the standard clinical lipid panel. For example, patients with reassuring non-HDL-C levels often have a discordantly high prevalence of LDL particles, which may portend greater atherosclerotic risk than one might expect from non-HDL-C alone [12]. Similarly, other advanced lipid tests could reveal risk that might not be obvious from the clinical lipids, which may explain why risk scoring systems making use of atherosclerosis measures outperform those based more on the clinical lipid assays [13]. Importantly, residual triglyceridemia may help identify patients who remain at risk from residual dyslipidemia, even though the triglycerides may be nowhere near the range raising concerns for pancreatitis. This raises interest in OM3-FAs, given their ability to improve residual dyslipidemia, motivating large clinical trials to determine whether they improve residual risk [11, 14].

OM3-ethyl esters (OM3-EE, Lovaza®, GlaxoSmithKline, Research Triangle Park, NC, USA) contain EPA and DHA as ethyl esters, which require hydrolysis by any of several lipases to be absorbed by the intestine, including pancreatic lipase [15]. In contrast, OM3-carboxylic acids (OM3-CA, Epanova®, AstraZeneca, Gaithersburg, MD, USA) are a complex mixture of EPA and DHA as free fatty acids (FFAs). Importantly, unlike ethyl esters, FFAs do not require hydrolysis by any of the lipases to be absorbed [15]. Both OM3-EE and OM3-CA significantly lower serum TG and are approved by the US Food and Drug Administration to treat patients with SHTG who are at risk of developing pancreatitis [16–18]. Studies in healthy volunteers indicate that OM3-CA achieves higher plasma exposure than OM3-EE under low-fat dietary conditions [19, 20]. This is consistent with the decrease in postprandial and interdigestive pancreatic lipase secretion that occurs when the relative portion of dietary fat is reduced [21]. This is important because it is expected that most patients with a history of pancreatitis and SHTG are on a low-fat diet [3, 8]. Perhaps lipase availability becomes so limiting on a chronic low-fat diet that the 2–4 g of added fat from OM3-EE is not hydrolyzed efficiently. In theory, this could cause delayed absorption, and in the extreme case, malabsorption of some portion of the dose load. Making matters worse, postprandial lipase secretion may prove even more limiting in patients with a history of pancreatitis due to pancreatic exocrine dysfunction [22, 23]. Divergent exposure profiles from different OM3-FA formulations is thus a relevant issue to consider in these patients. Our hypothesis was that the plasma exposure from EPA and DHA as ethyl esters (OM3-EE) would be lower relative to EPA and DHA as free carboxylic fatty acids (OM3-CA) in patients with SHTG and a history of acute pancreatitis who are on a low-fat diet. To test this hypothesis, we evaluated the plasma exposure of OM3-CA and OM3-EE under low-fat dietary conditions in the seldom-studied population with a history of hospital-treated pancreatitis with SHTG as its presumed cause.

## Methods

### Study design

**Fig. 1:**
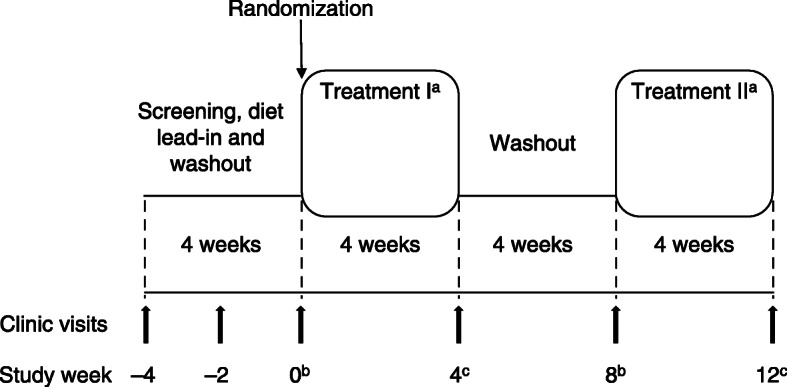
Crossover study design. Details of the assessments at each visit are provided in the methods. ^a^Treatment sequences were: OM3-CA 4 g/day: OM3-EE 4 g/day (*n* = 4); OM3-EE 4 g/day: OM3-CA 4 g/day (*n* = 4); OM3-CA 2 g/day: OM3-EE 4 g/day (*n* = 3); and OM3-EE 4 g/day: OM3-CA 2 g/day (*n* = 4). ^b^Participants were given a 4-week supply of the study drug and took their first dose. ^c^Pharmacokinetics assessment visit. Study dose was administered in combination with a liquid meal providing 500 kcal (12 g fat, 18 g protein and 80 g carbohydrates). Blood samples were collected before (*t* = 0) the low-fat load, and 1, 2, 3, 4, 5, 6, 7, 8, 9, 10, 12 and 24 h afterwards for postprandial assessments of omega-3 fatty acids, TGs, FFAs and apolipoproteins. Participants received breakfast, lunch, dinner and snacks (according to their calculated energy needs for weight maintenance) for consumption in the 2 days before the pharmacokinetics visit. OM3-CA, omega-3 carboxylic fatty acids; OM3-EE, omega-3 ethyl esters

The ECLIPSE IV study (NCT02189252) was a randomized, open-label, phase 1, crossover study conducted in patients with SHTG and a previous event of acute pancreatitis that was caused by SHTG. The study was conducted between October 1, 2014 and July 21, 2015 at six clinical sites in the United States and Canada. The aim was to compare, under low-fat dietary conditions, the 24-h plasma exposure of OM3-CA 2 g and OM3-CA 4 g with that of OM3-EE 4 g. The 24-h plasma exposure was assessed on the last day of 4 weeks of treatment with the same respective formulation and dosage (with a prescribed low-fat diet), by which time plasma EPA and DHA trough concentrations were expected to be at steady state based on a previous healthy volunteer study [20]. Each crossover sequence consisted of two 4-week treatment periods separated by a 4-week washout period (Fig. 1). Patients on OM3-CA during the first treatment period crossed over onto OM3-EE for the second period, and vice versa.

The exposure from 28 days daily dosing was assessed in terms of 24-h pharmacokinetics (PK) measures on day 28 of plasma total EPA and DHA (referred to in the text as EPA + DHA from this point on), free EPA and DHA, and ethyl esters of EPA and DHA. OM3-CA contains approximately 550 mg of EPA and 200 mg of DHA per gram and OM3-EE contains 465 mg of EPA and 375 mg of DHA per gram. However, the molar content of EPA + DHA is nearly identical per gram of OM3-CA (2.44 mmol) and OM3-EE (2.46 mmol), making the molar concentration the most appropriate measure for assessing differences in exposure from these formulations. Therefore, the PK measures of primary interest were baseline (day 0)-adjusted area under the plasma concentration versus time curve, from time 0 (where *t* = 0 is the start of liquid meal consumption and intake of the study drug) to the last measurable concentration (AUC_0–24_) and baseline-adjusted maximum measured plasma concentration over the time span specified (*C*_max_), for the molar concentration of plasma EPA + DHA. In line with the aims of the current study, baseline was defined as day 0, before patients received their first dose of study treatment. This was done to account for the expected accumulation over time (with the daily dosing regimen) of pre-dose/trough EPA and DHA levels in the 24-h AUC assessment at day 28. Importantly, this was done in a similar way to the ECLIPSE II trial in healthy volunteers [20]. Thus, the current study was designed to match the prior study so results could be compared and contrasted across disease status. Because of the unique patient population (SHTG and history of pancreatitis) and limited background information regarding expected effect sizes and variance, we lacked sufficient information to support a plausible formal power calculation. To illustrate the scientific problem, it is important to consider that EPA and DHA distribute in the plasma almost entirely as components of lipoproteins. Therefore, EPA and DHA pharmacokinetics are inextricably linked to lipoprotein kinetics. By definition, patients suffering SHTG have severely deranged lipoprotein metabolism, often involving impaired catabolism of the TG-rich lipoproteins. Accordingly, we could not place reasonable confidence on power calculations based on non-SHTG populations, insofar as they lack severely disordered lipoprotein metabolism that would alter the kinetics of lipoprotein components, including plasma EPA + DHA incorporated into lipoproteins. Another way of saying this is that one would expect a disease X PK interaction. If so, results from non-diseased subjects would not provide a plausible guide for a diseased population. Thus, we cannot place confidence in power calculations. Accordingly, we consider the results of this study exploratory, providing some of the first data on this seldom studied yet clinically important population.

### Participants

To be eligible for study entry, individuals had to be at least 18 years of age, have a history of SHTG (TG ≥ 500 mg/dL [5.6 mmol/L]) in the past 5 years and have at least one documented episode of hospitalization for acute pancreatitis due to SHTG. Major exclusion criteria were pregnancy, poorly controlled hypertension, serious comorbidity, conditions that could prevent compliance with the study protocol, gastrointestinal disorders that could affect absorption of the study drug, and allergy to the study drug or standardized diets. Details of exclusion criteria are given in Additional file 1, Supplemental Box S1.

### Study procedures

Prior to initiation of the phase 1, crossover study, the clinical study protocol was reviewed and approved by the institutional review board or independent ethics committee for each of six study centers in the USA and Canada. The study was conducted in compliance with the ethical principles in the Declaration of Helsinki and applicable amendments, and the International Conference on Harmonisation Good Clinical Practice guidelines. All participants provided written, informed consent. Individuals meeting the eligibility criteria at visit 1 (week − 4) entered a 4-week screening/diet lead-in/washout period (Fig. 1). During this period, and for the remainder of the study, individuals were not allowed to consume fish or use OM3-FA medications or supplements, meal replacement products, or to take weight-loss supplements or medications (including pancreatic lipase inhibitors), pancreatic enzyme preparations or systemic corticosteroids. Participants were prescribed the NCEP TLC diet with a caloric target for weight maintenance, or a low-fat diet [8]. Eligible patients provided a stool sample for measurement of fecal elastase-1, an accepted measure of exocrine pancreas function [24].

Assessments at visit 1 (week − 4) and visit 2 (week − 2) of the washout period consisted of body weight, vital signs, assessment of eligibility criteria and medical history. Screening assessments at visit 2 consisted of physical examination, electrocardiogram, urinalysis, serum chemistry, hematology and measurement of glycated hemoglobin concentrations. After the washout period, the 15 participants were randomized in a 1:1:1:1 ratio to the following treatment sequences:

1. OM3-CA 4 g once daily (4 weeks) **→** OM3-EE 4 g once daily (4 weeks) (*n* = 4)
2. OM3-EE 4 g once daily (4 weeks) **→** OM3-CA 4 g once daily (4 weeks) (*n* = 4)
3. OM3-CA 2 g once daily (4 weeks) **→** OM3-EE 4 g once daily (4 weeks) (*n* = 3)
4. OM3-EE 4 g once daily (4 weeks) **→** OM3-CA 2 g once daily (4 weeks) (*n* = 4)

At visit 3 (week/day 0, baseline), participants were given a 4-week supply of the study drug and took their first dose. Blood samples were taken 1.5, 0.75 and 0.25 h before the first dose of study drug for baseline assessment of OM3-FAs. Other assessments performed at visit 3 (and on week 4/day 28 of taking study drug) included fasting serum lipids and other biomarkers, body weight, vital signs, serum chemistry, hematology, blood viscosity, fibrinogen and recording of adverse events (AEs). Participants received a cooler at home containing breakfast, lunch, dinner and snacks on day 26 (according to their calculated energy needs for weight maintenance) to be consumed in the 2 days before visit 4 (week 4/day 28). At visit 4 (week 4/day 28), they were administered the study drug in combination with a liquid, low-fat meal providing 500 kcal (12 g fat, 18 g protein and 80 g carbohydrates). The meal was consumed over a 20-min period. Study drug was consumed after one-third of the meal had been ingested. Blood samples were collected before the liquid meal (*t* = 0) and at regular intervals (Fig. 1) up to 24 h afterward, for postprandial assessments of OM3-FAs, TG, FFA and apolipoproteins. A standardized, low-fat, EPA- and DHA-free lunch and dinner were provided 30 min after 5- and 10-h blood samples were taken, respectively. All blood samples were collected in K_2_EDTA tubes via venipuncture or intravenous catheter and stored at − 70 °C for biochemical analyses. After visit 4, participants entered a second washout period, prior to entering treatment period II. Study visits and assessments in treatment period II mirrored those of treatment period I.

### Biochemical assays

Assays for PK, pharmacodynamic and safety assessments were performed by Medpace Bioanalytical Laboratories and Medpace Reference Laboratories (Cincinnati, OH, USA). Plasma concentrations of OM3-FAs were measured by liquid chromatography-tandem mass spectrometry. OM3-FA, TG, FFA, apolipoproteins, blood viscosity and other biomarker and safety assessments used in this study have been used previously and are generally accepted as reliable, accurate and relevant to the evaluation of lipid-altering medications [7, 8, 19, 25–29]. Measurement of fecal elastase-1 was done using a pancreatic elastase-1 enzyme-linked immunosorbent assay stool test (ScheBo, Biotech AG, Giessen, Germany). Fecal elastase-1 is determined within the range of 15–500 μg per gram of stool. A fecal elastase-1 value of at least 200 μg/g of stool indicates normal pancreatic exocrine function, and a value less than 200 μg/g could indicate pancreatic exocrine insufficiency [24].

### Statistical methods

This was an exploratory study. Baseline adjustments for the calculation of AUC_0–24_ and *C*_max_ for comparisons of OM3-FA exposure between treatments were performed by subtracting the mean of the three pre-treatment concentrations (drawn prior to the start of each 4-week/28-day treatment period) from each sequential post-dose concentration during the 24-h assessment period.

Correcting for a baseline that is 28 days before the PK assessment has important implications for the interpretation of the acute-on-chronic exposure data. Because OM3-FAs are accumulating in plasma over the 28 days, our method for calculating incremental parameters reflects two drug effects: 1) the chronic incremental rise in c-trough from days 0 through 28 and 2) the superimposed incremental rise following the acute dose on day 28. Both increments make important contributions to exposure. As such, rather than split them, we decided a priori to analyze them as an aggregated quantity. As before, this approach maintains consistency with the prior study of healthy subjects, facilitating comparison. This omnibus measure can be considered a double increment, combining the chronic increment with the acute-on-chronic increment. Again, working in this rare population also presented problems powering the study; accordingly, splitting the increment into finer quantities seemed difficult to justify at this exploratory stage of the research.

Incremental PK parameters were compared between treatments on their natural log scales using a linear mixed model (LMM), with treatment, treatment period and treatment sequence as fixed effects and subject (treatment sequence) as a random effect. For fasting serum lipids and other biomarkers, average concentrations at the 4-week treatment endpoint were compared between treatments using LMM as above, with the addition that respective baseline values in their natural log scales were also adjusted as a fixed effect. Treatment-specific least-squares means (LSMs) and their differences were estimated from the above models. These estimates were then back-transformed to obtain geometric LSMs, percentage geometric LSM ratios (GLSMRs) with OM3-EE as the reference, and associated 95% confidence intervals and *P* values.

Isolated postprandial treatment effects on TG, FFA and apolipoprotein concentrations (pre-meal/dose-adjusted AUC_0–24_ [Pma-AUC_0–24_] and *C*_max_ [Pma-*C*_max_]) were originally planned to be analyzed using the same LMM model as above, as per the statistical protocol. However, a higher than expected number of post-meal/dose measures taken for these metabolites were below pre-meal/dose levels, resulting in negative values that could not be log-transformed for inclusion in the LMM. To avoid this issue, Pma-AUC_0–24_ and Pma-*C*_max_ values were instead compared between treatments using a non-parametric signed ranked test. Consistent with this analysis method, postprandial treatment effect data are presented as medians (interquartile range).

Descriptive data were presented as arithmetic means (standard deviation [SD]) and medians (range) unless otherwise specified. All analyses were performed using Phoenix® WinNonlin® Version 6.3 and/or SAS® Version 9.3 (SAS Institute, Inc. Cary, NC, USA). AEs were coded to system organ class and preferred term using the Medical Dictionary for Regulatory Activities (Version 17.0).

## Results

### Baseline characteristics and participant flow

In total, 15 patients with SHTG and a history of hospitalization for acute pancreatitis caused by SHTG were randomized: most were men under 65 years of age and were taking both lipid-lowering and diabetic medications (Table 1). The mean baseline fasting plasma TG concentration across all treatments was 10.0 (SD: 11.6) mmol/L and the median baseline fasting plasma TG concentration was 6.7 (interquartile range: 3.5–9.4) mmol/L. One patient who received OM3-EE 4 g followed by OM3-CA 2 g had a very high baseline fasting TG value of 48.7 mmol/L. Contrary to a priori expectations, only one participant had a fecal elastase-1 result (18 μg/g) consistent with pancreatic exocrine insufficiency. No patients had a history of chronic pancreatitis, but one patient with a fecal elastase-1 level of 396 μg/g had a pancreatic pseudocyst at the time of the study. Two patients discontinued, both in treatment sequence 2, during the first treatment period (one with an AE and one who withdrew consent for other reasons), both of whom were included in the intention-to-treat analysis. Of the 13 participants who completed the study, one was excluded from the OM3-FA PK analyses owing to consumption of protocol-prohibited OM3-FA-containing medication.

**Table 1:** Baseline demographic and patient characteristics

Variable	*N* = 15 *n* (%)
Age, years	
Mean ± SD	49.8 ± 11.2
< 65	14 (93.3)
≥ 65	1 (6.7)
Women	5 (33.3)
Body mass index, kg/m^2^	
Mean ± SD	33.0 (7.4)
18.5–24.9	2 (13.3)
25.0–29.9	3 (20.0)
≥ 30.0	10 (66.7)
Ethnicity	
White	14 (93.3)
Other	0 (0.0)
Triglycerides, mmol/L	
Mean ± SD	9.3 ± 9.4
Median (range)	5.8 (3.1, 39.3)
Total cholesterol, mmol/L	
Mean ± SD	5.4 (1.8)
Median (range)	5.0 (3.2, 8.4)
Lipid-lowering medication	
Fibrates	14 (93.3)
Statins	12 (80.0)
Cholesterol absorption inhibitors	3 (20.0)
History of diabetes	
Type 1	1 (6.7)
Type 2	11 (73.3)
Any	12 (80.0)
Diabetes medication	
Metformin	9 (60.0)
Insulin	2 (13.3)
Thiazolidinediones	2 (13.3)
Dipeptidyl peptidase-4 inhibitor	1 (6.7)
Alpha-glucosidase inhibitor	1 (6.7)
Fecal elastase, μg/g	
*n*	14 (93.3)
Median μg/g (range)	380 (18–500)

Data are for the safety population, defined as all participants who took at least one dose of study drug (*N* = 15). *SD* standard deviation

### OM3-FA exposure

**Fig. 2:**
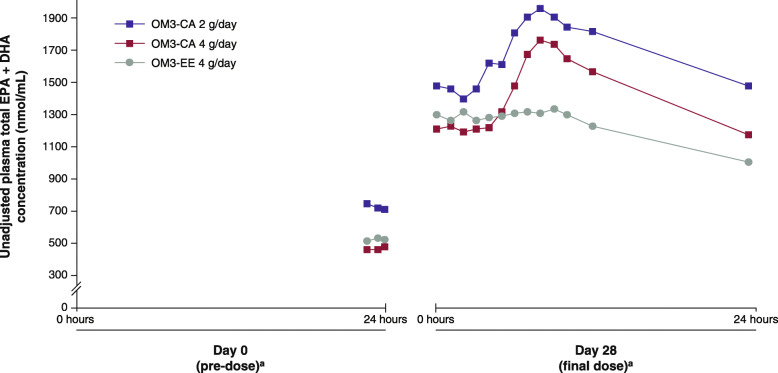
Mean unadjusted plasma total EPA + total DHA concentrations over the whole study. ^a^Mean values are for Treatment I and Treatment II (see Fig. 1)

Mean fasting plasma EPA + DHA concentrations (nmol/mL) for the pre-dose samples (− 1.5, − 0.75 and − 0.25 h) taken at the baseline visits (day 0 of Treatment I [study week 0] and Treatment II [study week 8]; Fig. 1) were 723 (SD, 465) for OM3-CA 2 g, 465 (SD, 305) for OM3-CA 4 g and 522 (SD, 260) for OM3-EE 4 g treatments (Fig. 2). After 4 weeks (day 28) of dosing while on a low-fat diet, mean pre-dose, fasting (pre-meal [*t* = 0]) plasma EPA + DHA concentrations had increased by similar amounts from day 0 for OM3-CA 2 g (+ 747 nmol/mL), OM3-CA 4 g (+ 735 nmol/mL) and OM3-EE 4 g (+ 768 nmol/mL) (Fig. 2).

**Fig. 3:**
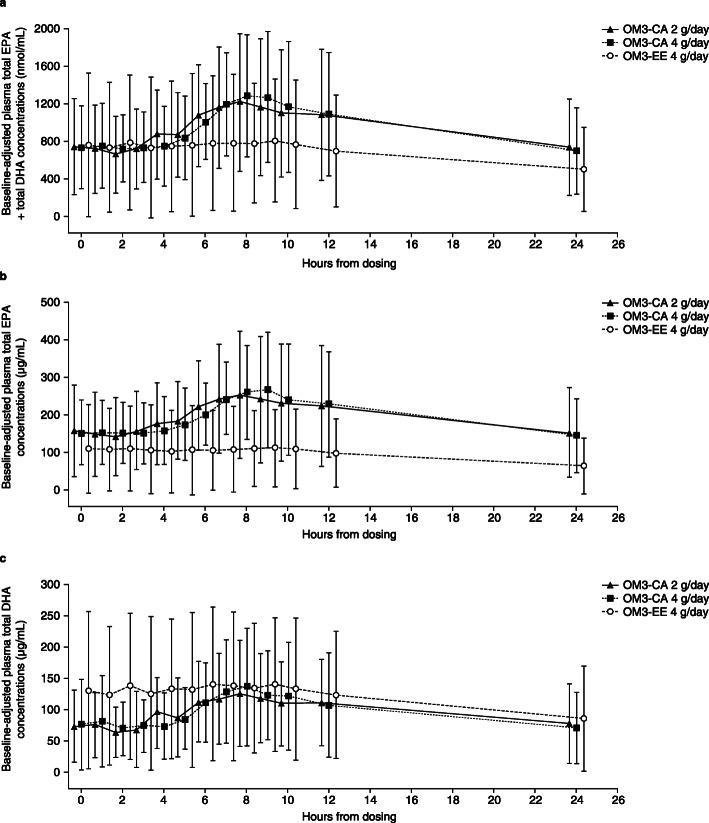
Mean (± SD) baseline-adjusted concentrations over time (24-h pharmacokinetic assessment on day 28 for Treatment I and Treatment II) for the different treatments administered with a low-fat liquid meal. (**a**) Plasma total EPA + total DHA, (**b**) total EPA and (**c**) total DHA for the OM3-CA 2 g/day (*n* = 6); OM3-CA 4 g/day (n = 6) and OM3-EE 4 g/day (*n* = 12) treatments. Blood samples were taken 1.5, 0.75 and 0.25 h before the first dose of study drug on day 0 (baseline). These values were then averaged and subtracted from each individual unadjusted concentration from 0 to 24 h to obtain baseline-adjusted values. DHA, docosahexaenoic acid; EPA, eicosapentaenoic acid; OM3-CA, omega-3 carboxylic acids; OM3-EE, omega-3 ethyl esters; SD, standard deviation

At the 4-week clinic visit (day 28), the study drug was ingested with the low-fat liquid meal for the 24-h acute-on-chronic exposure (PK) assessment. The mean maximal increase in plasma EPA + DHA concentrations (*C*_max_) from pre-dose (*t* = 0) concentrations was substantial for the OM3-CA 2 g and OM3-CA 4 g treatments, increasing by + 480 (+ 32.7%) and + 550 (+ 45.8%) nmol/mL to reach mean baseline-adjusted *C*_max_ values (at 8 h) of 1227 and 1285 nmol/mL, respectively (Fig. 3a). In contrast, mean plasma EPA + DHA did not substantially increase acutely after ingesting OM3-EE 4 g with a low-fat liquid meal, rising by + 40 nmol/mL (+ 3.1%) from the pre-dose/pre-meal concentration to reach a mean baseline-adjusted *C*_max_ value (at 9 h) of 808 nmol/mL. Similar patterns were seen when data for plasma EPA (Fig. 3b) and DHA (Fig. 3c) were analyzed separately, with little change from pre-meal concentrations occurring after OM3-EE 4 g ingestion compared with substantial increases after ingestion of OM3-CA 2 g and OM3-CA 4 g.

#### OM3-FA PK outcomes

Based on estimated GLSMRs, mean baseline (day 0)-adjusted AUC_0–24_ and *C*_max_ values for plasma EPA + DHA were numerically higher for OM3-CA 2 g versus OM3-EE 4 g (by + 22% and + 33%, respectively) and for OM3-CA 4 g versus OM3-EE 4 g (by + 60% and + 94%, respectively). However, these differences were not statistically significant (Table 2).

**Table 2:** Summary of statistical comparisons of baseline (day-0)-adjusted pharmacokinetic parameters between the treatment groups

PK parameter	Baseline-adjusted geometric LSMs at week-4 endpoint			Treatment comparison				Intra-subject CV (%)
	OM3-CA 2 g (*n* = 6)	OM3-CA 4 g (*n* = 6)	OM3-EE 4 g (*n* = 12)	OM3-CA 2 g vs OM3-EE 4 g		OM3-CA 4 g vs OM3-EE 4 g		
				%GLSMR (95% CI)	*P* value	%GLSMR (95% CI)	*P* value	
Total EPA + total DHA								
AUC_0–24_ (hour*nmol/mL)	14,431.18	18,992.50	11,866.94	121.6 (49.2, 300.8)	0.637	160.1 (64.7, 395.8)	0.27	75.6
*C*_max_ (nmol/mL)	916.66	1333.25	687.56	133.3 (68.5, 259.5)	0.354	193.9 (99.6, 377.5)	0.05	52.7
*T*_max_ (hour)	7.19	7.51	6.37	112.9 (66.3, 192.3)	0.618	118.0 (70.8, 196.6)	0.48	38.9
Total EPA								
AUC_0–24_ (hour*μg/mL)	2916.91	4248.45	1639.14	178.0 (82.2, 385.2)	0.126	259.2 (119.8, 561.0)	0.021	62.4
*C*_max_ (μg/mL)	181.32	289.40	96.82	187.3 (103.0, 340.7)	0.042	298.9 (164.3, 543.7)	0.003	46.7
*T*_max_ (hour)	6.57	7.32	6.45	101.9 (56.8, 183.1)	0.943	113.5 (63.2, 203.8)	0.636	42.9
Total DHA								
AUC_0–24_ (hour*μg/mL)	1451.24	1659.71	2091.28	69.4 (20.1, 240.0)	0.522	79.4 (23.0, 274.5)	0.683	115.7
*C*_max_ (μg/mL)	100.59	130.80	124.83	80.6 (34.6, 188.0)	0.578	104.8 (44.9, 244.4)	0.903	69.7
*T*_max_ (hour)	7.33	7.66	6.24	117.5 (71.0, 194.4)	0.488	122.7 (75.6, 199.0)	0.362	36.7

Comparisons were made between treatments of exposure (pharmacokinetic) parameters for baseline-adjusted plasma total EPA + total DHA, total EPA and total DHA. Means are geometric least-squares means (LSMs). Analysis is for the pharmacokinetics population, defined as all participants who completed both treatment periods and who had sufficient quantifiable plasma concentration data to calculate *C*_max_ (*n* = 12). Percentage geometric least squares mean ratio (%GLSMR) = 100*(Test/Reference). *CI* confidence interval, *CV* coefficient of variation, *DHA* docosahexaenoic acid, *EPA* eicosapentaenoic acid, *AUC*_*0–24*_ baseline-adjusted area under the plasma concentration versus time curve, from time 0 to 24 h after the start of the meal, C_max_ baseline-adjusted maximum measured plasma concentration over the time span specified, *OM3-CA* omega-3 carboxylic acids, *OM3-EE* omega-3 ethyl esters, *PK* pharmacokinetics, *T*_max_ time of the maximum measured plasma concentration

Estimated GLSMRs indicated that baseline-adjusted AUC_0–24_ and *C*_max_ values for plasma EPA were 159% and 199% higher, respectively, for OM3-CA 4 g than for OM3-EE 4 g, and these differences were statistically significant (Table 2). Greater plasma exposure of EPA was also observed for the lower OM3-CA 2 g dose compared with OM3-EE 4 g, with estimated GLSMRs indicating 78% and 87% higher baseline-adjusted AUC_0–24_ and *C*_max_ values, respectively; the difference was statistically significant for *C*_max_ (Table 2). The exposure of DHA (baseline-adjusted AUC_0–24_) was numerically slightly lower for OM3-CA 2 g and OM3-CA 4 g than for OM3-EE 4 g, but these differences were not statistically significant; nor was *C*_max_ significantly affected (Table 2). *T*_max_ (time of the maximum measured plasma concentration) values for plasma EPA + DHA, EPA and DHA were similar across all treatments, ranging from 6.2 to 7.7 h (Table 2).

Plasma concentrations of free EPA and DHA were very low (< 1% of total plasma levels; data not shown) and were therefore not analyzed. Similarly, ethyl esters of EPA and DHA were below the lower limit of quantification in the vast majority of samples (data not shown).

### TG, FFA and apolipoprotein assessments

On day 28, prior to receiving study treatment with the liquid low-fat meal, mean fasting plasma TG levels across the treatment arms were 5.7–15.8 mmol/L and mean fasting plasma FFA levels were 0.8–2.4 mmol/L (Additional file 1, Supplemental Table S1). Mean postprandial TG concentrations were flat for all three treatments during the 24-h PK assessment, indicating the low-fat liquid meal, containing 12 g of fat, had insufficient fat content to provoke a postprandial lipid peak (Additional file 1, Supplemental Figure S1). There were also no notable changes over time in concentrations of FFA, Apo A-I, Apo B-48, Apo B-100 or Apo C-III across the three treatments (Additional file 1, Supplemental Figure S2–S6). No significant postprandial differences in Pma-AUC_0–24_ or Pma-*C*_max_ were observed between treatments for TG, FFA or the apolipoproteins assessed (Additional file 1, Supplemental Tables S2–S7).

### Fasting serum lipids and biomarkers

Numerical reductions in fasting serum concentrations of TG, total cholesterol, very-low-density lipoprotein cholesterol (VLDL-C) and non-HDL-C from baseline to 4 weeks were observed for OM3-CA 2 g, OM3-CA 4 g and OM3-EE 4 g (Table 3 [mean, SD]; Additional file 1, Supplemental Table S8 [median, range]). No statistically significant differences between OM3-CA and OM3-EE were observed in terms of their effects on these measures (Additional file 1, Supplemental Table S9). There were no notable changes in other fasting serum biomarkers (remnant-like particle cholesterol, lipoprotein(a), high-sensitivity C-reactive protein, lipoprotein-associated phospholipase A2, arachidonic acid, adiponectin and leptin) from baseline to 4 weeks for OM3-CA or OM3-EE (data not shown).

**Table 3:** Summary of mean fasting serum lipid concentrations at baseline and after 4 weeks by treatment. Analysis is for the modified intent-to-treat population, defined as all participants who received at least one dose of study drug and provided at least one post-randomization efficacy value

Lipid parameter	OM3-CA 2 g (*n* = 7)	OM3-CA 4 g (*n* = 6)	OM3-EE 4 g (*n* = 14^a^)
Fasting TG (mmol/L)			
Baseline			
Mean (SD)	22.4 (36.2)	8.5 (5.7)	9.6 (10.0)
4-week endpoint			
Mean (SD)	15.8 (18.3)	5.7 (3.3)	8.2 (9.0)
Mean change from baseline (SD)	−6.6 (18.6)	−2.8 (3.2)	−1.4 (4.1)
Mean % change from baseline (SD)	−1.0 (41.0)	−25.6 (28.7)	−7.8 (40.0)
Fasting TC (mmol/L)			
Baseline			
Mean (SD)	7.2 (3.8)	4.9 (1.1)	5.6 (1.8)
4-week endpoint			
Mean (SD)	5.8 (2.2)	4.5 (0.8)	4.9 (1.4)
Mean change from baseline (SD)	−1.5 (1.8)	−0.4 (0.7)	−0.7 (1.2)
Mean % change from baseline (SD)	−15.9 (11.2)	−6.2 (11.0)	−9.3 (16.6)
Fasting direct LDL-C (mmol/L)			
Baseline			
Mean (SD)	1.5 (0.9)	1.5 (0.8)	1.8 (0.8)
4-week endpoint			
Mean (SD)	1.5 (0.7)	1.8 (0.5)	1.9 (1.0)
Mean change from baseline (SD)	−0.1 (0.2)	0.3 (0.6)	0.1 (0.5)
Mean % change from baseline (SD)	21.2 (71.6)	36.9 (51.6)	6.0 (29.4)
Fasting HDL-C (mmol/L)			
Baseline			
Mean (SD)	0.6 (0.2)	0.6 (0.1)	0.6 (0.2)
4-week endpoint			
Mean (SD)	0.5 (0.2)	0.6 (0.1)	0.6 (0.2)
Mean change from baseline (SD)	−0.04 (0.14)	0.05 (0.04)	−0.02 (0.12)
Mean % change from baseline (SD)	−2.7 (24.7)	8.3 (7.3)	−1.0 (21.1)
Fasting VLDL-C (mmol/L)			
Baseline			
Mean (SD)	5.1 (4.7)	2.8 (1.5)	3.2 (2.3)
4-week endpoint			
Mean (SD)	3.8 (2.8)	2.1 (1.1)	2.5 (1.7)
Mean change from baseline (SD)	−1.3 (2.0)	−0.7 (0.8)	−0.7 (1.2)
Mean % change from baseline (SD)	−17.8 (15.2)	−21.8 (22.5)	−13.3 (33.5)
Fasting non-HDL-C (mmol/L)			
Baseline			
Mean (SD)	6.7 (4.0)	4.3 (1.2)	5.0 (1.9)
4-week endpoint			
Mean (SD)	5.2 (2.4)	3.9 (0.9)	4.3 (1.5)
Mean change from baseline (SD)	−1.4 (1.8)	−0.4 (0.7)	−0.6 (1.3)
Mean % change from baseline (SD)	−16.3 (12.9)	−7.9 (11.7)	−9.7 (18.9)
Fasting TC:HDL-C			
Baseline			
Mean (SD)	19.0 (22.3)	9.0 (3.1)	9.9 (4.7)
4-week endpoint			
Mean (SD)	14.2 (11.6)	7.7 (1.9)	9.0 (4.5)
Mean change from baseline (SD)	−4.8 (12.9)	−1.3 (1.9)	−0.9 (2.8)
Mean % change from baseline (SD)	−6.8 (32.2)	−12.6 (12.7)	−4.6 (26.9)

^a^One patient on treatment sequence OM3-EE 4 g: OM3-CA 4 g discontinued from the study during treatment period I with valid lipid values measured at an unscheduled post-baseline visit. Therefore, this patient was included in treatment period I under OM3-EE 4 g but not in period II under OM3-CA 4 g. *HDL-C* high-density lipoprotein cholesterol, *LDL-C* low-density lipoprotein cholesterol, *OM3-CA* omega-3 carboxylic acids, *OM3-EE* omega-3 ethyl esters, *SD* standard deviation, *TC* total cholesterol, *TG* triglyceride, *VLDL-C* very-low-density lipoprotein cholesterol

### Viscosity measures

Measurements of fibrinogen (mean baseline value: 10.88 μmol/L; SD: 3.69) and blood viscosity under high-shear (mean baseline value: 3.89 cP; SD: 0.75) and low-shear (mean baseline value: 11.79 cP; SD: 11.79) conditions were available in 14 patients. No statistically significant changes from baseline were observed for these variables, including when hematocrit levels were normalized to 45% for the blood viscosity analyses (data not shown).

### Safety and tolerability

AE rates were low overall and similar for all three treatments (Table 4). No meaningful differences in clinical laboratory parameters were observed. Most treatment-emergent AEs were mild in severity, and no serious AEs or deaths from AEs occurred. Diarrhea (reported by four patients) was the only drug-related AE reported by more than one patient. One discontinuation occurred due to an AE of anemia in a patient with a history of bone marrow transplant; the anemia was mild in severity and deemed unrelated to the study drug (OM3-EE) by the investigator. One patient withdrew informed consent (treatment was OM3-EE).

**Table 4:** Number of patients experiencing adverse events (AEs) by treatment

AE category	Treatment		
	OM3-CA 2 g (*n* = 7) *n* (%)	OM3-CA 4 g (*n* = 6) *n* (%)	OM3-EE 4 g (*n* = 15) *n* (%)
Treatment-emergent AEs^a^	5	5	6
Mild	4	3	5
Moderate	1	2	1
Severe	0	0	0
Led to discontinuation	0	0	0
Related to study drug^b^	3	2	1
Diarrhea	1	2	0
Nausea	1	0	0
Abdominal distention	0	0	1
Abdominal pain	0	1	0
Dyspepsia	1	0	0
Somnolence	0	0	1
SAEs	0	0	0

Data are for the safety population, defined as all participants who took at least one dose of study drug (*n* = 15). ^a^A treatment-related AE was defined as any that started during treatment period I or II, or that was ongoing from the screening/washout period and subsequently worsened. ^b^The number of patients experiencing drug-related treatment-emergent AEs does not always equal the total number of different types of AEs by system organ class and preferred terms because more than one type of AE could be experienced by the same patient. *OM3-CA* omega-3 carboxylic acids, *OM3-EE* omega-3 ethyl esters, *SAE* serious adverse event

## Discussion

This was an exploratory, randomized, open-label, phase 1, crossover study, conducted in 15 patients with SHTG and a well-documented history of hospitalization for acute pancreatitis with SHTG as the presumed cause. Participants were given daily single doses of OM3-CA 2 g or OM3-CA 4 g (4.9 and 9.7 mmol of EPA + DHA as carboxylic acids, respectively) and OM3-EE 4 g (9.8 mmol of EPA + DHA as ethyl esters) for 4 weeks, as an adjunct to a structured, predefined low-fat diet. On day 28, the EPA + DHA exposure of each treatment was assessed over 24 h after ingestion of the last dose with a liquid low-fat meal.

A numerically greater, though not statistically significant, acute-on-chronic exposure was observed for OM3-CA 4 g than for OM3-EE 4 g in terms of EPA + DHA for baseline-adjusted AUC_0–24_ (1.6-fold higher) and *C*_max_ (1.9-fold) values. However, significantly greater exposure for OM3-CA 4 g compared with OM3-EE 4 g was observed when EPA was analyzed separately, with baseline-adjusted AUC_0–24_ being 2.6-fold higher and *C*_max_ being 3.0-fold higher. Despite these generous differences, these results should be interpreted with appropriate caution since they were exploratory and not adjusted for multiplicity. In addition, the EPA content of OM3-CA capsules is approximately 18% higher than for OM3-EE capsules. It is noteworthy that, despite approximately 90% higher DHA content in OM3-EE 4 g (1500 mg as ethyl esters) compared with OM3-CA 4 g (800 mg as carboxylic acids), the DHA plasma exposure was similar, consistent with enhanced plasma exposure from OM3-CA versus OM3-EE. Contrary to expectations [30], only one patient had a fecal elastase-1 concentration low enough to indicate potentially reduced pancreatic exocrine function. Low dietary fat, rather than pancreatic exocrine insufficiency, was presumably the dominant factor determining the relative exposure profiles of OM3-EE and OM3-CA.

The 24-h PK assessment clearly showed greater plasma exposure for EPA + DHA for OM3-CA (2 g and 4 g) than for OM3-EE 4 g (Figs. 2 and 3), consistent with our hypothesis and the results from the healthy volunteer study [20]. However, contrary to our hypothesis and the results of the healthy volunteer study, these acute-on-chronic exposure differences did not translate into higher fasting, pre-dose/trough plasma EPA + DHA concentrations with ‘chronic’ (4 weeks daily) dosing (Fig. 2).

It is not understood why trough exposure is dissociated from acute exposure. One reason for this may be poor adherence to the low-fat diet during the 4-week dosing period, which would lead to an insufficient reduction in pancreatic lipase secretion to observe a cumulative exposure benefit for OM3-CA over OM3-EE. Participants in the healthy volunteer study were confined to the clinical research unit during the entire dosing period, with meals prepared by study staff to ensure adherence to a low-fat diet [20]. In the current study, patients were confined to the clinic only for the 24-h PK assessment. Another potential explanation relates to pathophysiological aspects of dyslipidemia in SHTG, which include impairments in peripheral FFA trapping, increased FFA fluxes from adipocytes to the liver and dysregulation of hepatic very-low-density lipoprotein (VLDL) production [31]. Notably, baseline fasting plasma EPA + DHA concentrations were three to four times higher in our population than in the healthy volunteer study, and were highly variable [20]. Incorporation of EPA + DHA into plasma lipids, which are (by definition) more abundant in patients with SHTG than in healthy individuals, would likely be enhanced in these patients. Greatly elevated fasting plasma EPA + DHA levels in patients with SHTG may reflect lipoprotein-borne EPA + DHA that is esterified into TGs and other lipids, such as phospholipids and cholesteryl esters. In the SHTG population, defective lipoprotein clearance may cause lipoprotein-borne EPA + DHA to accumulate, just as other lipoprotein-borne esterified fatty acids accumulate. Interestingly, DHA was the main driver of the elevated baseline plasma EPA + DHA levels observed in our population, consistent with the preferential esterification of DHA into TGs [32]. It is therefore possible that elevated plasma DHA is, to some extent, a characteristic of patients with SHTG. Another contributing factor may be past fish-oil use, which has long been recommended and well adhered to in this population [33]. For this reason, we did not exclude patients with SHTG who were long-term fish oil users, lest we render the study un-enrollable. Instead, we washed participants out for 4 weeks. It is conceivable that over years or decades of exposure to fish oils, whole-body EPA + DHA may accumulate, mainly in adipose tissues, and possibly in ectopic fat depots, which may be released to plasma. Accordingly, we speculate that EPA and DHA behave like other fatty acids, insofar as fatty acid turnover in these tissues may contribute EPA + DHA to plasma for longer than 4 weeks after dietary intake of EPA + DHA ceases. The possibility of low compliance during the washout period also cannot be ruled out as a contributing source of EPA + DHA. These factors, alone or in combination with each other, may have caused the elevated and highly variable plasma EPA + DHA levels observed at baseline in our study. This could have obfuscated our ability to detect differences in trough levels of plasma EPA + DHA. We had speculated trough levels would be higher in OM3-CA owing to enhanced gut absorption of OM3-CA versus OM3-EE. As a caveat, we did not attempt to measure gut absorption in this experiment, a very onerous undertaking that involves detailed stool studies. Given the apparent disconnect between our mechanistic hypothesis based around altered gut absorption and comparable plasma exposures achieved at trough, it may help to undertake gut absorption studies in future experiments to resolve this.

Knowledge about the effects of lipid-lowering therapies on the risk of acute pancreatitis is emerging. A recent meta-analysis of 16 placebo-controlled and standard care-controlled statin trials (*N* = 113,800) with a mean follow-up of 4.1 years found a significant 23% (*P* = 0.03) reduction in the relative risk of acute pancreatitis with statin use [34]. In contrast, a borderline significant increase of 39% (*P* = 0.053) in the relative risk of acute pancreatitis was seen with fibrate use across seven trials (*N* = 40,162) with a mean follow-up period of 5.3 years. A major limitation of the phase 3 and 4 trials that comprise this analysis is the exclusion of people at high risk of pancreatitis from alcohol, gallstones, and SHTG itself, making it hard to extrapolate the apparent risk/benefit analysis to the SHTG population. Nevertheless, Preiss and Sattar extrapolated these data to SHTG, and offer that even statins could be justified as a first-line therapy for reducing pancreatitis risk in patients with SHTG [35]. Ultimately, widely varying approaches reflect the need for more research targeting the SHTG population in particular, a major motivation for the present work with OM3-CA. To wit, OM3-CA has been shown to be efficacious and to have an excellent safety profile (consistent with the pharmacodynamic and safety assessments in the current study), significantly reducing both TG and non-HDL-C in patients with SHTG [17]. Similar effects occur on top of statin therapy in patients with hypertriglyceridemia [18] along with significant drops in VLDL/chylomicron remnant prevalence and apolipoprotein C-III levels, consistent with complementary effects on TG metabolism beyond statin monotherapy [36]. Given the positive benefit/risk profile, combining a statin with OM3-FA treatment may be a useful additional option for reducing the risk of acute pancreatitis. Our 24-h PK results suggest acute-on-chronic plasma exposure from OM3-CA is greater than for OM3-EE under strictly controlled, low-fat dietary conditions, especially for EPA. To the extent that enhancing acute plasma exposure translates to enhanced TG-lowering, this may benefit SHTG patients. Specifically, in patients with SHTG, who are expected to be on a low-fat diet, OM3-CA (with or without a statin) may reduce the risk of acute pancreatitis to a greater extent than OM3-EE. Larger studies would be warranted to test this concept.

Interestingly, the liquid, low-fat meal given to patients prior to their final dose on day 28 evidently had insufficient fat to provoke any appreciable postprandial lipemia. Though this rendered the latter analyses inconclusive, it also gives a sense for how much fat must be given to prompt a detectable postprandial TG response, that is, well over 12 g dietary fat plus the fat from the OM3 preparations. Indeed, from a medical standpoint, our post-meal lipid profiles were excellent, and often lower than the pre-meal concentrations, which accords with expected benefits of fat-restriction in the SHTG population. These results raise an interesting clinical question—if we know a patient has consumed a low-fat meal, then may we rely on non-fasting TGs? Intriguingly, in the extreme case of SHTG, consuming a low-fat meal seems to yield almost identical results to fasting conditions. This is consistent with previous findings in the general population, where plasma TG changes minimally in response to normal food. Moreover, non-fasting lipid profiles better predict cardiovascular risk [37]. As a caveat, our postprandial analysis may not have had sufficient power to detect postprandial effects, especially as there was wide variability in TGs on day 28 prior to the postprandial assessments. It is also possible that the absence of an increase in postprandial TG is due, to some extent, to ongoing treatment with OM-3-CA or OM-3-EE, which have been shown to reduce postprandial TGs [32], although this is a purely speculative proposition given the absence of a placebo control group in our study.

In parallel with our work, OM3-FAs are also being developed for an indication well outside of SHTG, namely, to reduce cardiovascular events. It is important to emphasize that their utility in atherosclerotic disease has no bearing on their utility in SHTG. These are distinct diseases with vastly different clinical outcomes and time courses, and the populations of these studies are for the most part non-overlapping. Given the persistent unmet medical need among SHTG patients, our results support further research aimed at reducing pancreatitis in SHTG.

## Conclusions

In conclusion, the present study provides the first data supporting the concept that patients with SHTG and a history of pancreatitis exhibit enhanced acute-on-chronic OM3 exposure from OM3-CA 4 g over OM3-EE 4 g under low-fat dietary conditions, especially for EPA. This unique patient group requires continual management of serum TG concentrations to prevent a recurrence of pancreatitis. OM3-CA, with its positive benefit/risk profile, may be a favorable option.

## Supplementary information

**Additional file 1 Supplemental Box S1** List of applied exclusion criteria. **Supplemental Table S1** Pre-meal/dose concentrations of TG, FFA and apolipoproteins on day 28. **Supplemental Table S2** Summary of statistical comparisons between treatments of postprandial pharmacodynamic (PD) parameters calculated from pre-meal/dose-adjusted (Pma) serum triglyceride values on day 28. **Supplemental Table S3** Summary of statistical comparisons between treatments of postprandial pharmacodynamic (PD) parameters calculated from pre-meal/dose-adjusted (Pma) serum free fatty acid values on day 28. **Supplemental Table S4** Summary of statistical comparisons between treatments of postprandial pharmacodynamic (PD) parameters calculated from pre-meal/dose-adjusted (Pma) serum Apo A-I values on day 28 . **Supplemental Table S5** Summary of statistical comparisons between treatments of postprandial pharmacodynamic (PD) parameters calculated from pre-meal/dose-adjusted (Pma) serum Apo B-48 values on day 28. **Supplemental Table S6** Summary of statistical comparisons between treatments of postprandial pharmacodynamic (PD) parameters calculated from pre-meal/dose-adjusted (Pma) serum Apo B-100 values on day 28 . **Supplemental Table S7** Summary of statistical comparisons between treatments of postprandial pharmacodynamic (PD) parameters calculated from pre-meal/dose-adjusted (Pma) serum Apo C-III values on day 28. **Supplemental Table S8** Summary of median fasting serum lipid concentrations at baseline and after 4 weeks by treatment . **Supplemental Table S9** Summary of statistical comparisons of baseline-adjusted fasting lipid concentrations between treatments. **Supplemental Figure S1** Mean (standard deviation) unadjusted postprandial triglyceride concentration over time. **Supplemental Figure S2** Mean (standard deviation) unadjusted postprandial free fatty acid concentration versus time. **Supplemental Figure S3** Mean (standard deviation) unadjusted postprandial Apo A-I concentration versus time. **Supplemental Figure S4** Mean (standard deviation) unadjusted postprandial Apo B-48 concentration versus time. **Supplemental Figure S5** Mean (standard deviation) unadjusted postprandial Apo B-100 concentration versus time. **Supplemental Figure S6** Mean (standard deviation) unadjusted postprandial Apo C-III concentration versus time.

## Data Availability

The datasets used and/or analyzed during the current study are available from the corresponding author on reasonable request.

## Abbreviations

AE: Adverse event
DHA: Docosahexaenoic acid
EPA: Eicosapentaenoic acid
GLSMR: Geometric least-squares mean ratio
HDL-C: High-density lipoprotein cholesterol
AUC_0–24_: Mean baseline-adjusted area under the plasma concentration versus time curve
*C*_max_: Baseline-adjusted maximum measured plasma concentration over the time span specified
LMM: Linear mixed model
LSM: Least-squares mean
NCEP TLC: National Cholesterol Education Program Therapeutic Lifestyle Changes
OM3-CA: Omega-3 carboxylic acids
OM3-EE: Omega-3 ethyl esters
OM3-FA: Omega-3 fatty acids
PK: Pharmacokinetics
Pma: Pre-meal/dose-adjusted
SD: Standard deviation
SHTG: Severe hypertriglyceridemia
TG: Triglyceride
*T*_max_: Time of the maximum measured plasma concentration
VLDL: Very-low-density lipoprotein
VLDL-C: Very-low-density lipoprotein cholesterol
