# Supplementary material for: Omega-3 fatty acid exposure with a low-fat diet in patients with past hypertriglyceridemia-induced acute pancreatitis; an exploratory, randomized, open-label crossover study
Source: Lipids Health Dis. 2020 May 30;19:117. doi: 10.1186/s12944-020-01295-7 (PMC7260759; doi:10.1186/s12944-020-01295-7)
Supplement: Supplementary file 1 — Additional file 1 Supplemental Box S1 List of applied exclusion criteria. Supplemental Table S1 Pre-meal/dose concentrations of TG, FFA and apolipoproteins on day 28. Supplemental Table S2 Summary of statistical comparisons between treatments of postprandial pharmacodynamic (PD) parameters calculated from pre-meal/dose-adjusted (Pma) serum triglyceride values on day 28. Supplemental Table S3 Summary of statistical comparisons between treatments of postprandial pharmacodynamic (PD) parameters calculated from pre-meal/dose-adjusted (Pma) serum free fatty acid values on day 28. Supplemental Table S4 Summary of statistical comparisons between treatments of postprandial pharmacodynamic (PD) parameters calculated from pre-meal/dose-adjusted (Pma) serum Apo A-I values on day 28 . Supplemental Table S5 Summary of statistical comparisons between treatments of postprandial pharmacodynamic (PD) parameters calculated from pre-meal/dose-adjusted (Pma) serum Apo B-48 values on day 28. Supplemental Table S6 Summary of statistical comparisons between treatments of postprandial pharmacodynamic (PD) parameters calculated from pre-meal/dose-adjusted (Pma) serum Apo B-100 values on day 28 . Supplemental Table S7 Summary of statistical comparisons between treatments of postprandial pharmacodynamic (PD) parameters calculated from pre-meal/dose-adjusted (Pma) serum Apo C-III values on day 28. Supplemental Table S8 Summary of median fasting serum lipid concentrations at baseline and after 4 weeks by treatment . Supplemental Table S9 Summary of statistical comparisons of baseline-adjusted fasting lipid concentrations between treatments. Supplemental Figure S1 Mean (standard deviation) unadjusted postprandial triglyceride concentration over time. Supplemental Figure S2 Mean (standard deviation) unadjusted postprandial free fatty acid concentration versus time. Supplemental Figure S3 Mean (standard deviation) unadjusted postprandial Apo A-I concentration versus time. Supplemental Figure S4 Me [file 12944_2020_1295_MOESM1_ESM.docx]

SUPPLEMENTAL INFORMATION

**OM3-FA bioavailability with low-fat diet in patients with past hypertriglyceridemia-induced acute pancreatitis; A randomized, open-label crossover study**

Richard L. Dunbar,^1,2,3*^ Daniel Gaudet,^4^ Michael Davidson,^5,6^ Hong Yang,^7^ Catarina Nilsson,^7^ Mats Kvarnström^7^ and Jan Oscarsson^7^

^1^Cardiometabolic and Lipid Clinic, Corporal Michael J. Crescenz VA Medical Center, Philadelphia, PA, USA

^2^Division of Translational Medicine and Human Genetics, Department of Medicine, Perelman School of Medicine at the University of Pennsylvania, Philadelphia, PA, USA

^3^ICON plc, North Wales, PA, USA

^4^Lipidology Unit, Community Genomic Medicine Centre and ECOGENE-21, Department of Medicine, Université de Montréal, Saguenay, QC, Canada

^5^University of Chicago Pritzker School of Medicine, Chicago, IL, USA

^6^Corvidia Therapeutics, Waltham, MA, USA

^7^AstraZeneca, Gothenburg, Sweden

**Supplemental Box S1** List of applied exclusion criteria

- - Allergy or intolerance to components of the study drugs, fish or components of the standardized meals; poorly controlled hypertension (resting blood pressure ≥ 160 mm Hg systolic and/or ≥ 100 mm Hg diastolic) before randomization.
  - Cancer (other than basal cell carcinoma) in the last 2 years.
  - Cardiovascular event, aortic aneurysm or resection, ceratoid endarterectomy or revascularization procedure in the 6 months before visit 1.
  - Clinically important endocrine, pulmonary, hepatic, renal, hematologic, immunologic, dermatologic, neurologic, psychiatric or biliary disorders in the 6 months before visit 1.
  - Gastrointestinal disorder in the 6 months before visit 1 that the investigator judged as having the potential to disrupt normal digestion and absorption.
  - An abnormal laboratory result at visit 2 judged by the investigator to be of clinical significance (one re-test was allowed at visit 3 for participants with an abnormal laboratory result).
  - Alcohol or substance abuse (> 14 drinks per week), or strong potential for abuse, in the 12 months before visit 1.
  - Pregnancy.
  - Exposed to the study drug in the 30 days before visit 1.
  - Any condition the investigator believed would interfere with the ability of participants to provide informed consent or compliance with the study protocol.

**Supplemental Table S1** Pre-meal/dose concentrations of TG, FFA and apolipoproteins on day 28. Data are for the modified intent-to-treat population, defined as all participants who received at least one dose of study drug and provided at least one post-randomization efficacy value

| Metabolite concentration | OM3-CA 2 g  *(n* = 7) | OM3-CA 4 g  *(n* = 6) | OM3-EE 4 g  *(n* = 13) |
| --- | --- | --- | --- |
|  |  |  |  |
| TG, mmol/L (pre-meal/dose) |  |  |  |
| Mean (SD) | 15.80 (18.32) | 5.67 (3.30) | 8.57 (9.24) |
| Median (range) | 11.11 (2.45, 55.05) | 4.33 (2.68, 10.83) | 5.05 (2.29, 36.07) |
| FFA, mmol/L (pre-meal/dose) |  |  |  |
| Mean (SD) | 2.351 (3.528) | 0.823 (0.262) | 1.498 (2.453) |
| Median (range) | 0.834 (0.292, 10.08) | 0.835 (0.433, 1.215) | 0.703 (0.296, 9.470) |
| Apo A-1, g/L (pre-meal/dose) |  |  |  |
| Mean (SD) | 1.02 (0.19) | 1.11 (0.10) | 1.10 (0.17) |
| Median (range) | 1.05 (0.72, 1.22) | 1.09 (0.98, 1.29) | 1.13 (0.76, 1.30) |
| Apo B-48, g/L (pre-meal/dose) |  |  |  |
| Mean (SD) | 0.0466 (0.0299) | 0.0244 (0.0145) | 0.0345 (0.0260) |
| Median (range) | 0.0573 (0.0085, 0.0815) | 0.0182 (0.0138, 0.0528) | 0.0236 (0.0102, 0.0882) |
| Apo B-100, g/L (pre-meal/dose) |  |  |  |
| Mean (SD) | 0.8739 (0.2610) | 0.9479 (0.1445) | 0.8745 (0.2342) |
| Median (range) | 0.8765 (0.4317, 1.2527) | 0.8978 (0.8242, 1.2135) | 0.8470 (0.3631, 1.3252) |
| Apo C-III, g/L (pre-meal/dose) |  |  |  |
| Mean (SD) | 0.2679 (0.1021) | 0.1885 (0.0790) | 0.2193 (0.0846) |
| Median (range) | 0.2634 (0.1287, 0.4140) | 0.1694 (0.1103, 0.2954) | 0.1948 (0.0862, 0.3622) |

Apo, apolipoprotein; FFA, free fatty acid; OM3-CA, omega-3 carboxylic acids; OM3-EE, omega-3 ethyl esters; SD, standard deviation; TG, triglyceride

**Supplemental Table S2** Summary of statistical comparisons between treatments of postprandial pharmacodynamic (PD) parameters calculated from pre‑meal/dose-adjusted (Pma) serum triglyceride values on day 28. Analysis is for the modified intent-to-treat population, defined as all participants who received at least one dose of study drug and provided at least one post-randomization efficacy value

| PD parameter | Median (IQR) | | | Treatment comparison | | | |
| --- | --- | --- | --- | --- | --- | --- | --- |
|  | OM3-CA 2 g  (*n* = 7) | OM3-CA 4 g  (*n* = 6) | OM3-EE 4 g  (*n* = 13) | OM3-CA 2 g vs OM3-EE 4 g | | OM3-CA 4 g vs OM3-EE 4 g | |
|  |  |  |  | Median difference (IQR) | *P* value | Median difference (IQR) | *P* value |
| Pma-AUC_0–24_ (hour*mmol/L) | 7.6 (–80.5, 20.6) | 3.5 (–0.73, 6.4) | 2.3 (–4.5, 8.7) | –0.11 (–52.37, 12.10) | 1.0000 | 0.12 (–2.29, 3.00) | 1.0000 |
| Pma-*C*_max_ (mmol/L) | 0.89 (–2.02, 2.01) | 0.95 (0.80, 1.26) | 0.74 (0.50, 1.08) | 0.41 (–2.27, 0.76) | 0.8125 | 0.24 (–0.10, 0.32) | 0.5625 |

AUC_0–24_, area under the plasma concentration versus time curve, from time 0 to 24 hours after the start of the meal; *C*_max_, maximum measured plasma concentration over the time span specified; IQR, interquartile range; OM3-CA, omega-3 carboxylic acids; OM3-EE, omega-3 ethyl esters

**Supplemental Table S3** Summary of statistical comparisons between treatments of postprandial pharmacodynamic (PD) parameters calculated from pre‑meal/dose-adjusted (Pma) serum free fatty acid values on day 28. Analysis is for the modified intent-to-treat population, defined as all participants who received at least one dose of study drug and provided at least one post-randomization efficacy value

| PD parameter | Median (IQR) | | | Treatment comparison | | | |
| --- | --- | --- | --- | --- | --- | --- | --- |
|  | OM3-CA 2 g  (*n* = 7) | OM3-CA 4 g  (*n* = 6) | OM3-EE 4 g  (*n* = 12) | OM3-CA 2 g vs OM3-EE 4 g | | OM3-CA 4 g vs OM3-EE 4 g | |
|  |  |  |  | Median difference (IQR) | *P* value | Median difference (IQR) | *P* value |
| Pma-AUC_0–24_ (hour*mmol/L) | –7.13 (–8.56, 1.56) | –7.22 (–12.79, –1.98) | –8.54 (–10.77, –4.68) | –0.06 (–0.42, 1.47) | 1.0000 | 2.35 (–1.88, 3.66) | 0.5625 |
| Pma-*C*_max_ (mmol/L) | 0.13 (–0.12, 0.97) | –0.08 (–0.22, 0.09) | –0.09 (–0.21, –0.01) | 0.02 (–0.07, 0.81) | 0.2969 | 0.08 (–0.05, 0.18) | 0.6875 |

AUC_0–24_, area under the plasma concentration versus time curve, from time 0 to 24 hours after the start of the meal; *C*_max_, maximum measured plasma concentration over the time span specified; IQR, interquartile range; OM3-CA, omega-3 carboxylic acids; OM3-EE, omega-3 ethyl esters

**Supplemental Table S4** Summary of statistical comparisons between treatments of postprandial pharmacodynamic (PD) parameters calculated from pre‑meal/dose-adjusted (Pma) serum Apo A-I values on day 28. Analysis is for the modified intent-to-treat population, defined as all participants who received at least one dose of study drug and provided at least one post-randomization efficacy value

| PD parameter | Median (IQR) | | | Treatment comparison | | | |
| --- | --- | --- | --- | --- | --- | --- | --- |
|  | OM3-CA 2 g  (*n* = 7) | OM3-CA 4 g  (*n* = 6) | OM3-EE 4 g  (*n* = 13) | OM3-CA 2 g vs OM3-EE 4 g | | OM3-CA 4 g vs OM3-EE 4 g | |
|  |  |  |  | Median difference (IQR) | *P* value | Median difference (IQR) | *P* value |
| Pma-AUC_0–24_ (g/L) | –1.38 (–2.18, –0.13) | –1.96 (–3.59, –1.55) | –1.51 (–1.82, –0.95) | –0.86 (–1.16, 0.49) | 0.4688 | –0.39 (–2.26, 2.10) | 0.8438 |
| Pma-*C*_max_ (g/L) | 0 (–0.01, 0.02) | –0.01 (–0.05, 0.04) | 0 (–0.01, 0.02) | –0.03 (–0.06, 0.01) | 0.5625 | 0.01 (–0.05, 0.04) | 0.8125 |

Apo, apolipoprotein; AUC_0–24_, area under the plasma concentration versus time curve, from time 0 to 24 hours after the start of the meal; *C*_max_, maximum measured plasma concentration over the time span specified; IQR, interquartile range; OM3-CA, omega-3 carboxylic acids; OM3-EE, omega-3 ethyl esters

**Supplemental Table S5** Summary of statistical comparisons between treatments of postprandial pharmacodynamic (PD) parameters calculated from pre‑meal/dose-adjusted (Pma) serum Apo B-48 values on day 28. Analysis is for the modified intent-to-treat population, defined as all participants who received at least one dose of study drug and provided at least one post-randomization efficacy value

| PD parameter | Median (IQR) | | | Treatment comparison | | | | | |
| --- | --- | --- | --- | --- | --- | --- | --- | --- | --- |
|  | OM3-CA 2 g  (*n* = 7) | OM3-CA 4 g  (*n* = 6) | OM3-EE 4 g  (*n* = 13) | OM3-CA 2 g vs OM3-EE 4 g | | | OM3-CA 4 g vs OM3-EE 4 g | | |
|  |  |  |  | Median difference (IQR) | *P* value | Median difference (IQR) | | *P* value |  |
| Pma-AUC_0–24_ (g/L) | 0.04 (–0.17, 0.14) | 0.12 (0.06, 0.14) | 0.07 (–0.01, 0.15) | 0.01 (–0.13, 0.19) | 0.8125 | 0.03 (–0.10, 0.07) | | 0.8438 |  |
| Pma-*C*_max_ (g/L) | 0.007 (0.005, 0.012) | 0.012 (0.009, 0.015) | 0.012 (0.006, 0.013) | 0.0003 (–0.009, 0.006) | 0.9375 | –0.0002 (–0.005, 0.006) | | 1.0000 |  |

Apo, apolipoprotein; AUC_0–24_, area under the plasma concentration versus time curve, from time 0 to 24 hours after the start of the meal; *C*_max_, maximum measured plasma concentration over the time span specified; IQR, interquartile range; OM3-CA, omega-3 carboxylic acids; OM3-EE, omega-3 ethyl esters

**Supplemental Table S6** Summary of statistical comparisons between treatments of postprandial pharmacodynamic (PD) parameters calculated from pre‑meal/dose-adjusted (Pma) serum Apo B-100 values on day 28. Analysis is for the modified intent-to-treat population, defined as all participants who received at least one dose of study drug and provided at least one post-randomization efficacy value

| PD parameter | Median (IQR) | | | | Treatment comparison | | | | | |
| --- | --- | --- | --- | --- | --- | --- | --- | --- | --- | --- |
|  | OM3-CA 2 g  (*n* = 7) | OM3-CA 4 g  (*n* = 6) | OM3-EE 4 g  (*n* = 13) | OM3-CA 2 g vs OM3-EE 4 g | | | OM3-CA 4 g vs OM3-EE 4 g | | |  |
|  |  |  |  | Median difference (IQR) | | *P* value | Median difference (IQR) | *P* value |  |  |
| Pma-AUC_0–24_ (g/L) | –0.60 (–0.89, –0.22) | –1.43 (–2.37, –0.94) | –0.63 (–0.88, –0.18) | –0.64 (–0.75, 0.16) | | 0.4688 | –0.21 (–1.75, 1.05) | 0.6875 |  |  |
| Pma-*C*_max_ (g/L) | 0.024 (0.022, 0.034) | 0.01 (–0.01, 0.04) | 0.04 (0.02, 0.05) | –0.03 (–0.05, 0.02) | | 0.2969 | –0.02 (–0.04, 0.03) | 0.6875 |  |  |

Apo, apolipoprotein; AUC_0–24_, area under the plasma concentration versus time curve, from time 0 to 24 hours after the start of the meal; *C*_max_, maximum measured plasma concentration over the time span specified; IQR, interquartile range; OM3-CA, omega-3 carboxylic acids; OM3-EE, omega-3 ethyl esters

**Supplemental Table S7** Summary of statistical comparisons between treatments of postprandial pharmacodynamic (PD) parameters calculated from pre‑meal/dose-adjusted (Pma) serum Apo C-III values on day 28. Analysis is for the modified intent-to-treat population, defined as all participants who received at least one dose of study drug and provided at least one post-randomization efficacy value

| PD parameter | Median (IQR) | | | Treatment comparison | | | |
| --- | --- | --- | --- | --- | --- | --- | --- |
|  | OM3-CA 2 g  (*n* = 7) | OM3-CA 4 g  (*n* = 6) | OM3-EE 4 g  (*n* = 13) | OM3-CA 2 g vs OM3-EE 4 g | | OM3-CA 4 g vs OM3-EE 4 g | |
|  |  |  |  | Median difference (IQR) | *P* value | Median difference (IQR) | *P* value |
| Pma-AUC_0–24_ (g/L) | –0.15 (–0.61, –0.12) | –0.12 (–0.30, –0.03) | –0.21 (–0.36, –0.04) | 0.17 (–0.19, 0.60) | 0.5781 | 0.05 (–0.01, 0.15) | 0.5625 |
| Pma-*C*_max_ (g/L) | 0.008 (0.003, 0.04) | 0.01 (0.003, 0.02) | 0.01 (–0.001, 0.02) | 0.013 (0.005, 0.03) | 0.0781 | 0.004 (–0.03, 0.01) | 0.8438 |

Apo, apolipoprotein; AUC_0–24_, area under the plasma concentration versus time curve, from time 0 to 24 hours after the start of the meal; *C*_max_, maximum measured plasma concentration over the time span specified; IQR, interquartile range; OM3-CA, omega-3 carboxylic acids; OM3-EE, omega-3 ethyl esters

**Supplemental Table S8** Summary of median fasting serum lipid concentrations at baseline and after 4 weeks by treatment. Analysis is for the modified intent-to-treat population, defined as all participants who received at least one dose of study drug and provided at least one post-randomization efficacy value

| Lipid parameter | OM3-CA 2 g  (*n* = 7) | OM3-CA 4 g  (*n* = 6) | OM3-EE 4 g  (*n* = 14^a^) |
| --- | --- | --- | --- |
|  |  |  |  |
| Fasting TG (mmol/L) |  |  |  |
| Baseline |  |  |  |
| Median (range) | 7.6 (3.2, 103.3) | 7.0 (3.2, 19.0) | 6.2 (2.7, 39.3) |
| 4-week endpoint |  |  |  |
| Median (range) | 11.1 (2.5, 55.1) | 4.3 (2.7, 10.8) | 5.0 (2.3, 36.1) |
| Median change from baseline (range) | –0.8 (–48.3, 5.7) | –1.6 (–8.2, 0.7) | –0.8 (–11.4, 7.0) |
| Median % change from baseline (range) | –9.2 (–46.7, 83.5) | –28.0 (–60.8, 20.6) | –13.5 (–51.3, 85.8) |
| Fasting TC (mmol/L) |  |  |  |
| Baseline |  |  |  |
| Median (range) | 6.7 (3.2, 14.0) | 4.6 (3.7, 6.3) | 5.8 (2.9, 8.4) |
| 4-week endpoint |  |  |  |
| Median (range) | 6.4 (2.9, 9.1) | 4.4 (3.5, 5.7) | 5.2 (2.7, 7.8) |
| Median change from baseline (range) | –0.6 (–4.9, –0.3) | –0.2 (–1.6, 0.3) | –0.3 (–3.2, 0.9) |
| Median % change from baseline (range) | –12.7 (–35.0, –4.7) | –4.3 (–25.9, 5.6) | –7.7 (–38.5, 16.1) |
| Fasting direct LDL-C (mmol/L) |  |  |  |
| Baseline |  |  |  |
| Median (range) | 1.4 (0.1, 2.9) | 1.4 (0.5, 2.5) | 1.9 (0.3, 3.3) |
| 4-week endpoint |  |  |  |
| Median (range) | 1.4 (0.3, 2.6) | 2.0 (0.8, 2.3) | 1.7 (0.3, 4.3) |
| Median change from baseline (range) | –0.1 (–0.4, 0.3) | 0.2 (–0.6, 1.1) | 0.1 (–0.7, 0.9) |
| Median % change from baseline (range) | –9.1 (–20.3, 180.0) | 30.4 (–22.1, 114.3) | 6.4 (–37.0, 54.8) |
| Fasting HDL-C (mmol/L) |  |  |  |
| Baseline |  |  |  |
| Median (range) | 0.6 (0.2, 0.9) | 0.6 (0.4, 0.6) | 0.6 (0.4, 1.1) |
| 4-week endpoint |  |  |  |
| Median (range) | 0.7 (0.3, 0.7) | 0.6 (0.5, 0.7) | 0.6 (0.3, 0.9) |
| Median change from baseline (range) | –0.1 (–0.2, 0.2) | 0.05 (0, 0.11) | 0 (–0.2, 0.2) |
| Median % change from baseline (range) | –14.1 (–27.8, 28.1) | 8.8 (0, 17.7) | 0 (–40.4, 38.3) |
| Fasting VLDL-C (mmol/L) |  |  |  |
| Baseline |  |  |  |
| Median (range) | 4.6 (0.9, 13.7) | 2.8 (1.2, 5.2) | 2.6 (0.9, 7.6) |
| 4-week endpoint |  |  |  |
| Median (range) | 4.1 (0.8, 8.6) | 2.0 (1.0, 3.3) | 1.7 (0.6, 6.3) |
| Median change from baseline (range) | –0.5 (–5.2, 0.1) | –0.5 (–1.9, 0.1) | –0.4 (–2.6, 1.5) |
| Median % change from baseline (range) | – 14.0 (–37.6, 1.7) | –21.1 (–55.7, 4.8) | –16.5 (–51.6, 70.3) |
| Fasting non-HDL-C (mmol/L) |  |  |  |
| Baseline |  |  |  |
| Median (range) | 6.0 (2.4, 13.8) | 4.0 (3.1, 5.7) | 5.0 (2.3, 7.9) |
| 4-week endpoint |  |  |  |
| Median (range) | 5.8 (2.3, 8.9) | 3.8 (2.9, 5.1) | 4.6 (2.0, 7.3) |
| Median change from baseline (range) | –0.4 (–5.0, –0.2) | –0.2 (–1.6, 0.3) | –0.3 (–3.2, 1.1) |
| Median % change from baseline (range) | –10.3 (–35.9, –2.5) | –6.6 (–28.6, 6.3) | –10.2 (–41.8, 23.2) |
| Fasting TC:HDL-C |  |  |  |
| Baseline |  |  |  |
| Median (range) | 9.9 (4.1, 67.8) | 7.9 (6.4, 14.8) | 8.7 (4.6, 16.7) |
| 4-week endpoint |  |  |  |
| Median (range) | 11.2 (4.4, 35.2) | 7.7 (5.7, 9.7) | 7.5 (4.0, 18.8) |
| Median change from baseline (range) | 0.2 (–32.6, 5.6) | –0.9 (–5.0, 0.5) | –0.7 (–5.8, 2.7) |
| Median % change from baseline (range) | 5.3 (–48.0, 29.1) | –12.3 (-–34.1, 5.7) | –10.9 (–38.4, 44.0) |

^a^One patient on treatment sequence OM3-EE 4 g: OM3-CA 4 g discontinued from the study during treatment period I with valid lipid values measured at an unscheduled post-baseline visit. Therefore, this patient was included in treatment period I under OM3-EE 4 g but not in period II under OM3-CA 4 g. HDL-C, high-density lipoprotein cholesterol; LDL-C, low-density lipoprotein cholesterol; OM3-CA, omega-3 carboxylic acids; OM3‑EE, omega-3 ethyl esters; TC, total cholesterol; TG, triglyceride; VLDL-C, very‑low-density lipoprotein cholesterol

**Supplemental Table S9** Summary of statistical comparisons of baseline-adjusted fasting lipid concentrations between treatments. Means are geometric least-squares means. Analysis is for the modified intent-to-treat population, defined as all participants who received at least one dose of study drug and provided at least one post-randomization efficacy value

| Lipid parameter | Baseline-adjusted geometric LSMs  at 4-week endpoint | | | Treatment comparison | | | |
| --- | --- | --- | --- | --- | --- | --- | --- |
|  | OM3-CA 2 g  (*n* = 7) | OM3-CA 4 g  (*n* = 6) | OM3-EE 4 g  (*n* = 14^a^) | OM3-CA 2 g vs OM3-EE 4 g | | OM3-CA 4 g vs OM3-EE 4 g | |
|  |  |  |  | %GLSMR  (95% CI) | *P* value | %GLSMR  (95% CI) | *P* value |
| Fasting TG (mmol/L) | 6.37 | 6.17 | 6.27 | 1.0 (0.7, 1.5) | 0.936 | 1.0 (0.6, 1.5) | 0.931 |
| Fasting TC (mmol/L) | 4.90 | 4.92 | 4.86 | 1.0 (0.9, 1.2) | 0.922 | 1.0 (0.9, 1.2) | 0.880 |
| Fasting direct LDL-C (mmol/L) | 1.74 | 1.46 | 1.49 | 1.2 (0.9, 1.5) | 0.245 | 1.0 (0.7, 1.3) | 0.873 |
| Fasting HDL-C (mmol/L) | 0.57 | 0.58 | 0.56 | 1.0 (0.8, 1.3) | 0.925 | 1.0 (0.8, 1.3) | 0.748 |
| Fasting VLDL-C (mmol/L) | 2.06 | 2.14 | 2.11 | 1.0 (0.7, 1.4) | 0.874 | 1.0 (0.7, 1.5) | 0.941 |
| Fasting non-HDL-C (mmol/L) | 4.25 | 4.24 | 4.23 | 1.0 (0.8, 1.2) | 0.944 | 1.0 (0.8, 1.2) | 0.971 |
| Fasting TC:HDL-C | 8.61 | 8.33 | 8.70 | 1.0 (0.7, 1.4) | 0.946 | 1.0 (0.7, 1.4) | 0.786 |

Percentage geometric least-squares mean ratio (%GLSMR) = 100*(Test/Reference). ^a^One patient on treatment sequence OM3-EE 4 g: OM3-CA 4 g discontinued from the study during treatment period I with valid lipid values measured at an unscheduled post-baseline visit. Therefore, this patient was included in treatment period I under OM3-EE 4 g but not in period II under OM3-CA 4 g. CI, confidence interval; HDL-C, high-density lipoprotein cholesterol; LDL-C, low-density lipoprotein cholesterol; LSM, least-squares mean; OM3-CA, omega-3 carboxylic acids; OM3-EE, omega-3 ethyl esters; TC, total cholesterol; TG, triglyceride; VLDL-C, very-low-density lipoprotein cholesterol

**
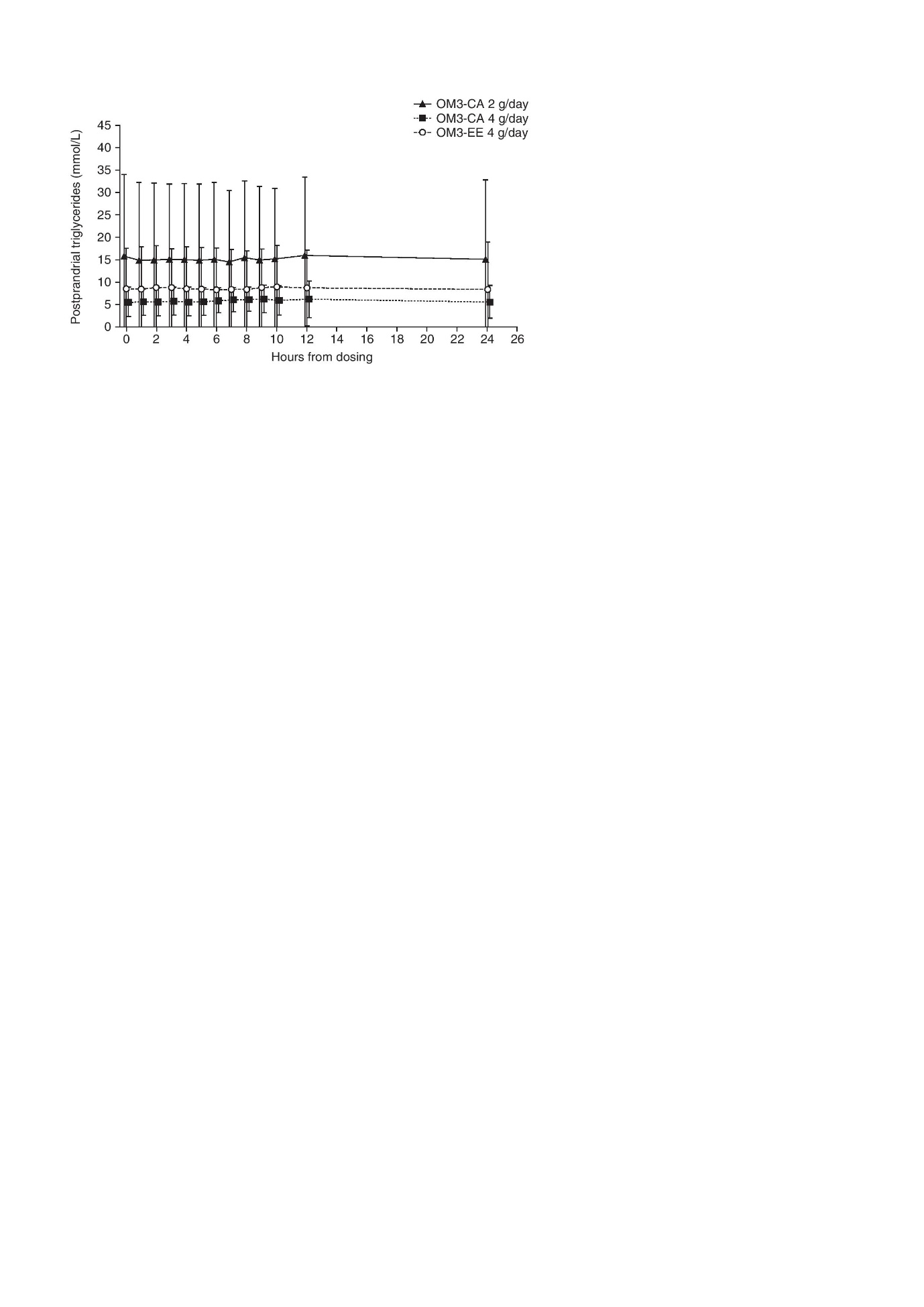
**

**Supplemental Fig. S1** Mean (standard deviation) unadjusted postprandial triglyceride concentration over time. Data are for the modified intent-to-treat population. OM3-CA, omega-3 carboxylic acids; OM3-EE, omega-3 ethyl esters

**
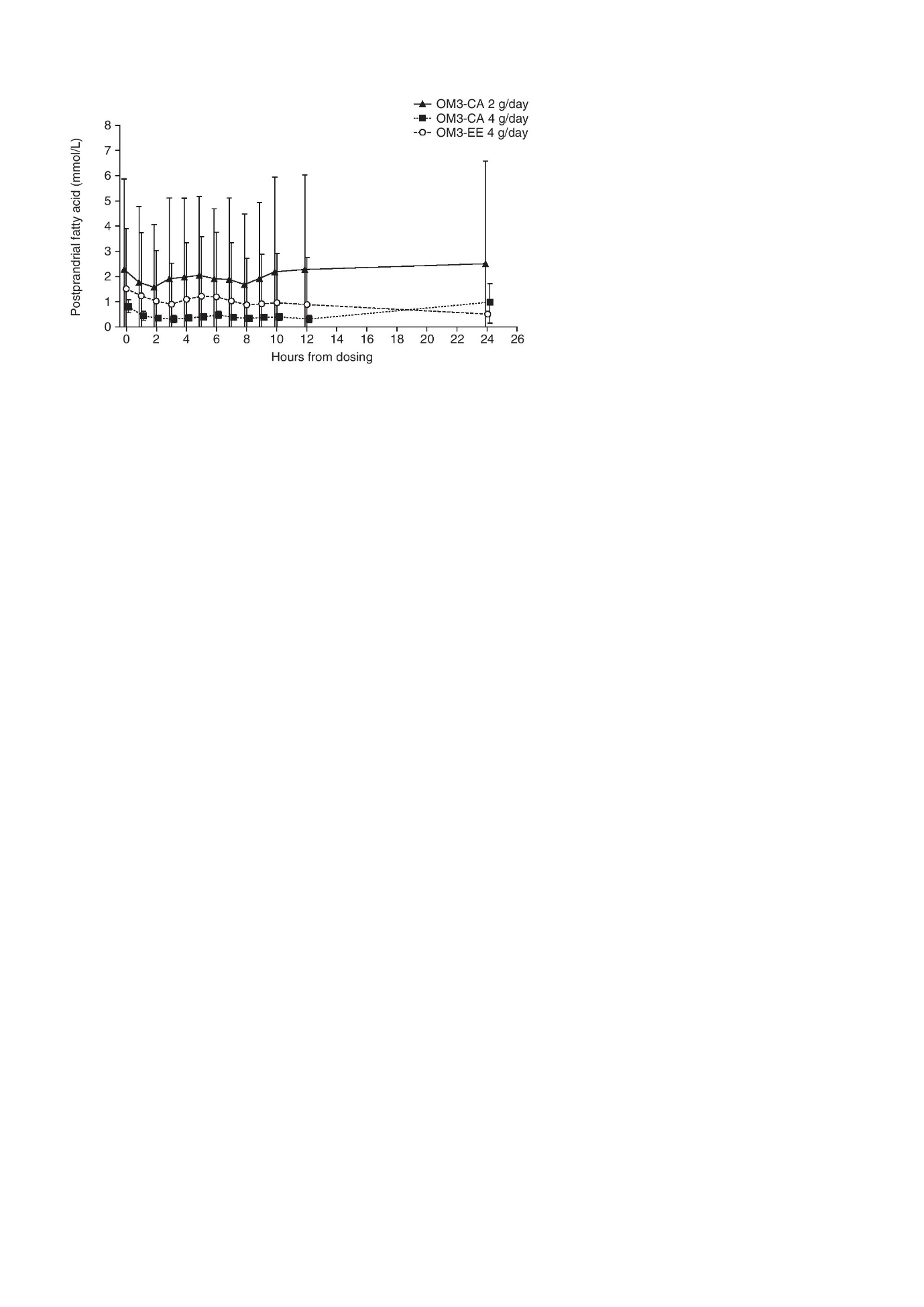
**

**Supplemental Fig. S2** Mean (standard deviation) unadjusted postprandial free fatty acid concentration versus time. Data are for the modified intent-to-treat population. OM3-CA, omega-3 carboxylic acids; OM3-EE, omega-3 ethyl esters


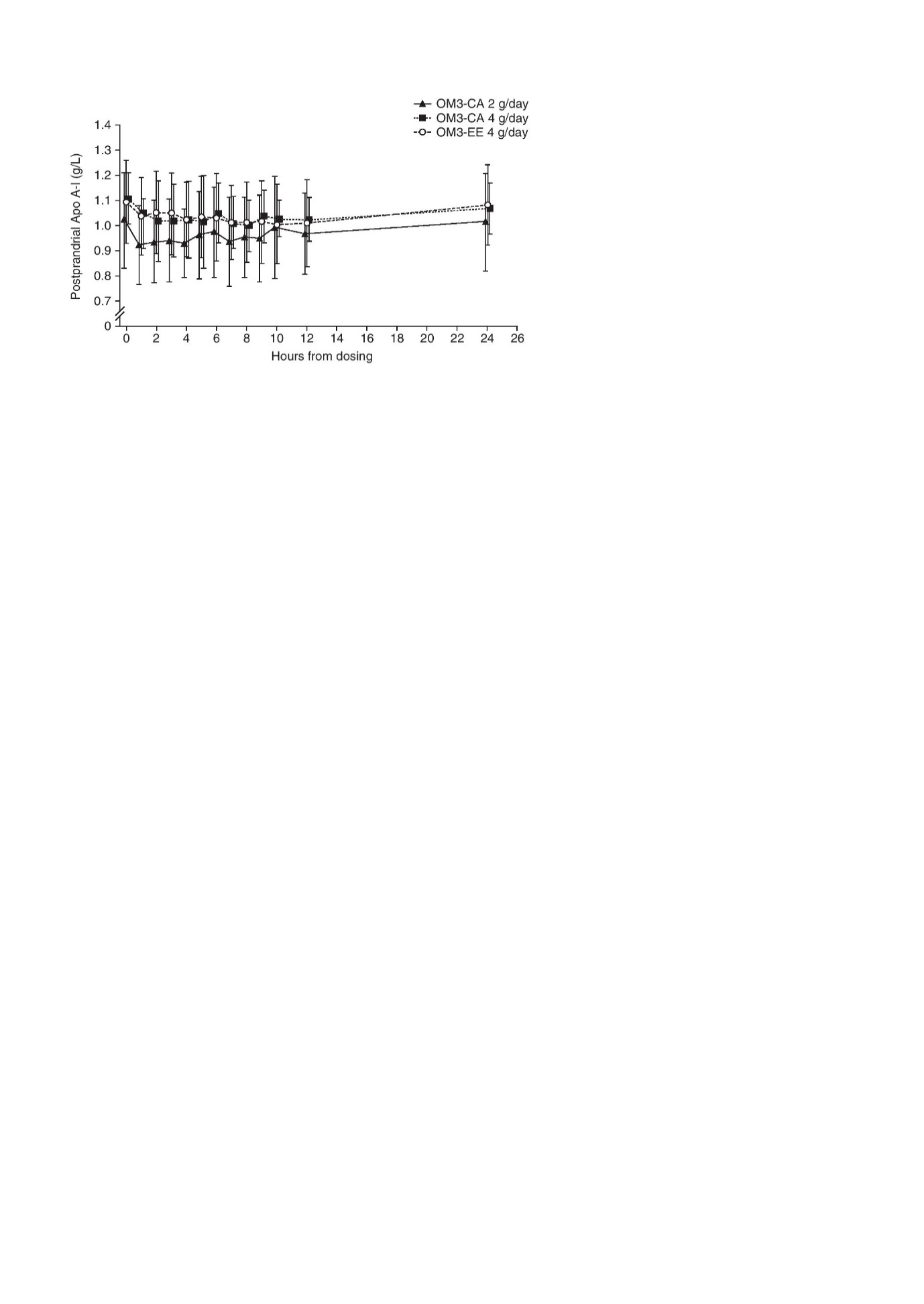


**Supplemental Fig. S3** Mean (standard deviation) unadjusted postprandial Apo A-I concentration versus time. Data are for the modified intent-to-treat population. Apo, apolipoprotein; OM3-CA, omega-3 carboxylic acids; OM3-EE, omega-3 ethyl esters

**
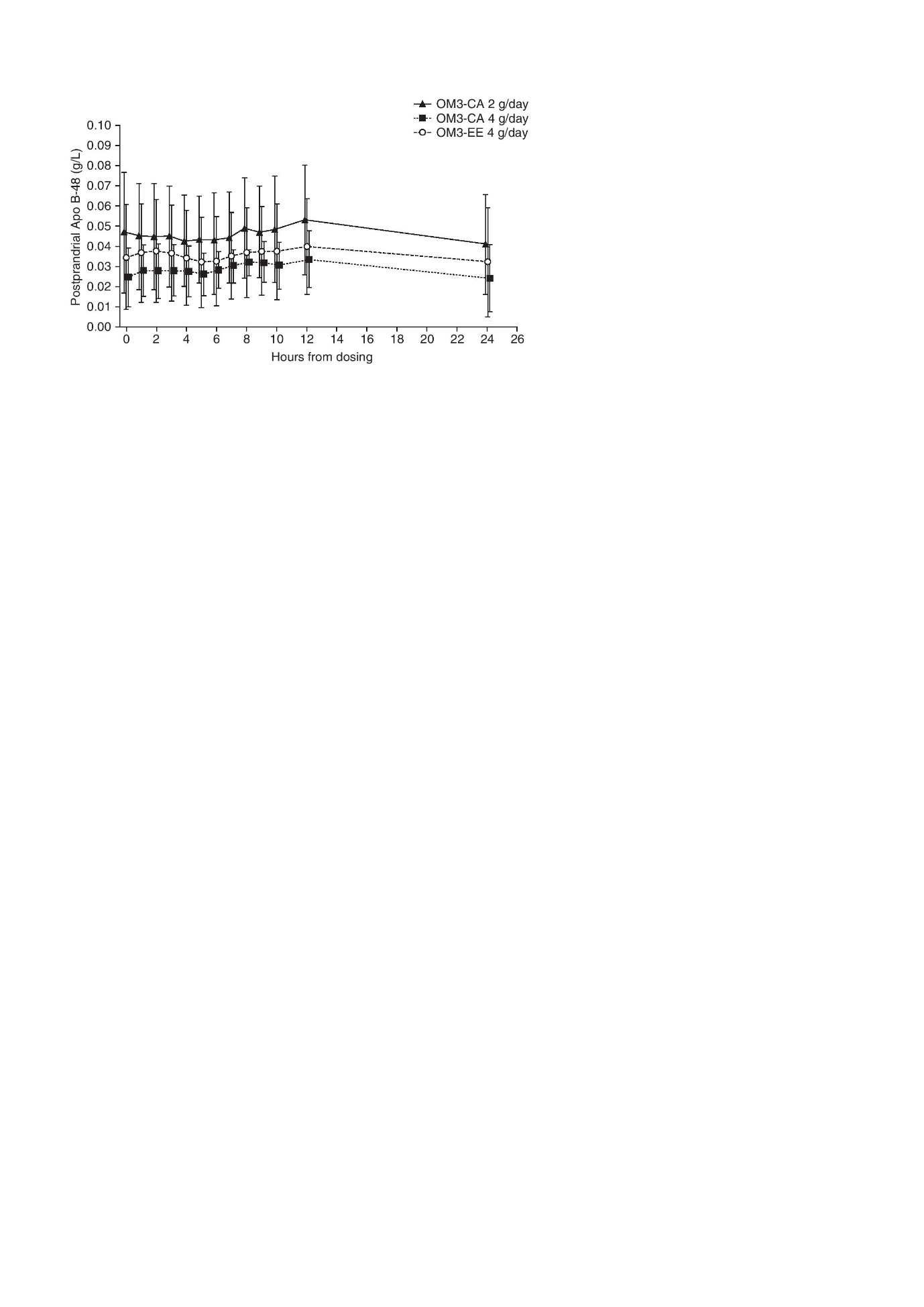
**

**Supplemental Fig. S4** Mean (standard deviation) unadjusted postprandial Apo B-48 concentration versus time. Data are for the modified intent-to-treat population. Apo, apolipoprotein; OM3-CA, omega-3 carboxylic acids; OM3-EE, omega-3 ethyl esters


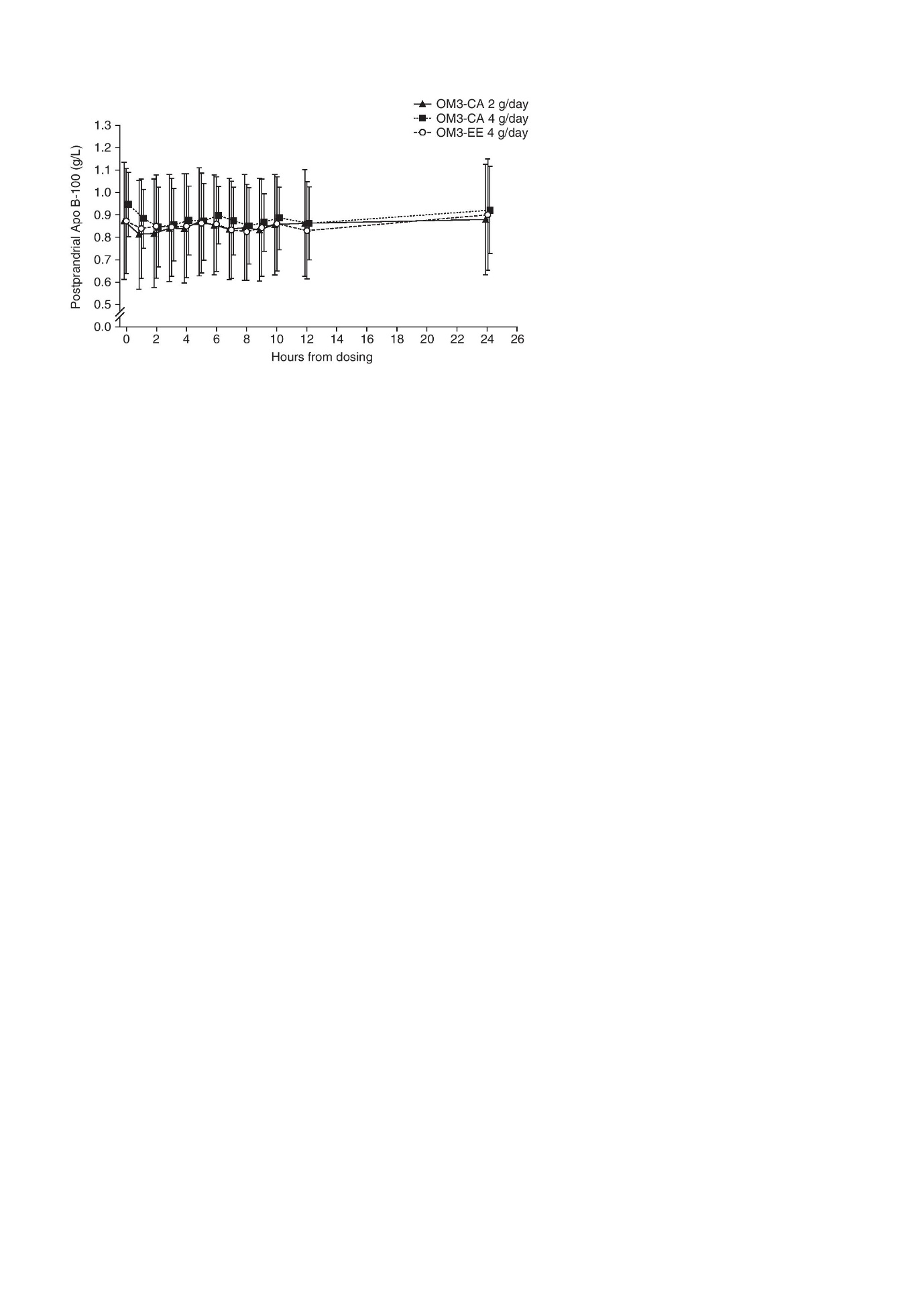


**Supplemental Fig. S5** Mean (standard deviation) unadjusted postprandial Apo B-100 concentration versus time. Data are for the modified intent-to-treat population. Apo, apolipoprotein; OM3-CA, omega-3 carboxylic acids; OM3-EE, omega-3 ethyl esters


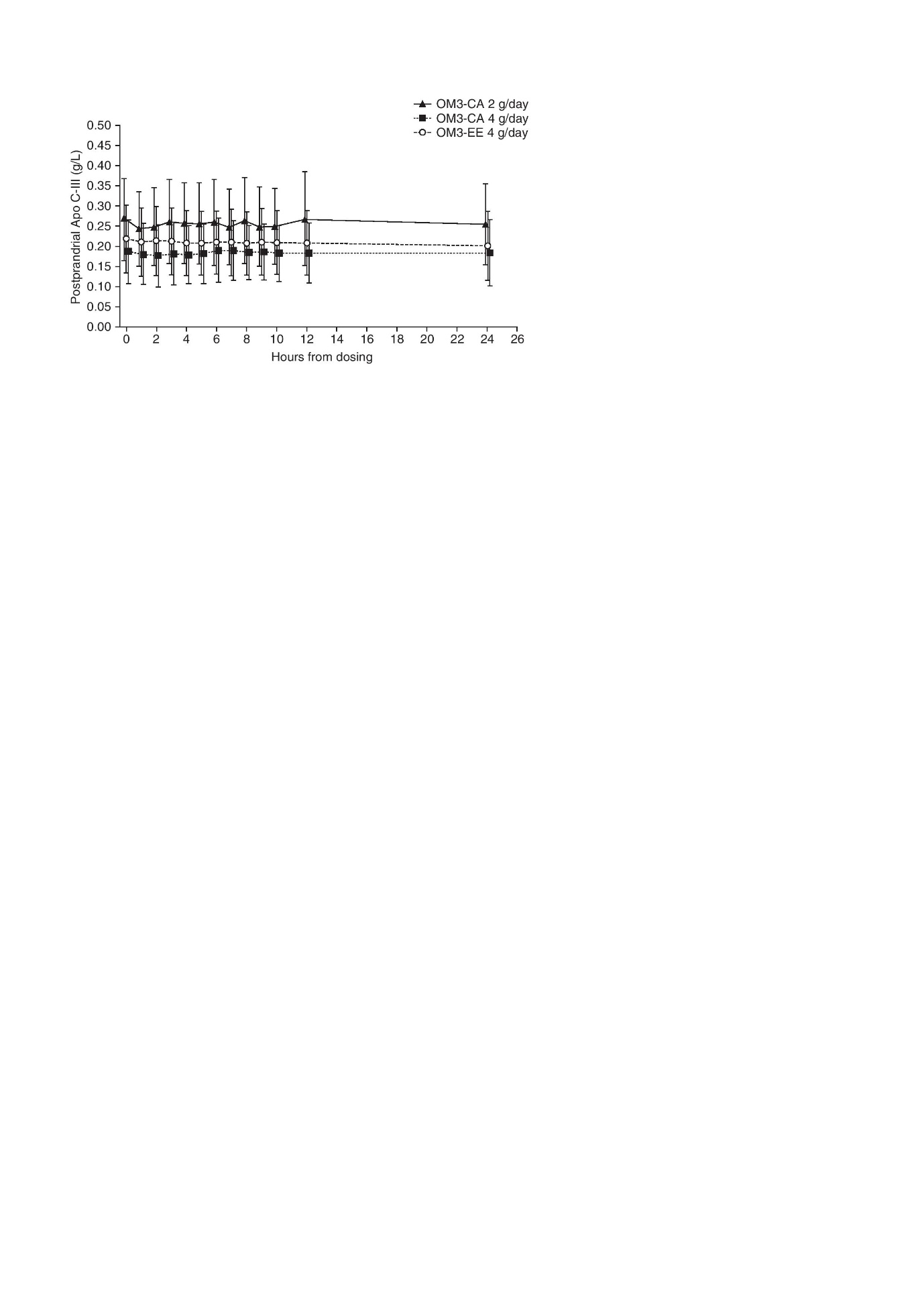


**Supplemental Fig. S6** Mean (standard deviation) unadjusted postprandial Apo C-III concentration versus time. Data are for the modified intent-to-treat population. Apo, apolipoprotein; OM3-CA, omega-3 carboxylic acids; OM3-EE, omega-3 ethyl esters
